# Gold Nanoparticles in Prostate Cancer: Advances in Targeted Therapy, Diagnostics, and Precision Nanomedicine

**DOI:** 10.3390/ph19081273

**Published:** 2026-08-12

**Authors:** Umme Hani, Mona Al Hamod, Noura Al Hamood, Yahya Alhamhoom, Mohammed Ghazwani, Fahad AlQahtani, Helal A. Helal, Riyaz Ali M. Osmani

**Affiliations:** 1 Department of Pharmaceutics, College of Pharmacy, King Khalid University, Abha 62223, Saudi Arabia; 2 Department of Pharmaceutics, Faculty of Pharmacy, Northern Border University, Rafha 73213, Saudi Arabia; 3 Department of Pharmaceutical Sciences, College of Pharmacy, Umm Al-Qura University, Makkah 21955, Saudi Arabia

**Keywords:** gold nanoparticles, prostate cancer, nanomedicine, photothermal therapy, radiosensitization, precision oncology

## Abstract

Prostate cancer (PC) is one of the most common cancers in men globally and there is an urgent need for new immune-based approaches because many traditional therapies, including chemotherapy, radiotherapy, and anti-androgens, have limitations. Due to their distinct physicochemical and biological features, gold nanoparticles (AuNPs) are emerging as a potential nanoplatform for the development of strategies in prostate cancer therapy. These features include tunable size, shape, and surface plasmon resonance (SPR) and high surface-to-volume ratio, which result in enhanced drug loading, targeted delivery and improved bioavailability. In addition, AuNPs can also be functionalized for active targeting to promote selective accumulation in tumors while minimizing systemic toxicity. In addition, the intrinsic optical and photothermal properties of these nanoparticles allow them to serve for PTT, radiosensitization and multimodal imaging, i.e., CT (computed tomography) and photoacoustic imaging. These have shown potential in pre-clinical and clinical evaluation but face issues with long-term toxicity, biodistribution and large-scale manufacturing. In this review, we summarize the organizing features, functional properties, therapeutic activities and translational potentials of AuNPs in prostate cancer treatment, with emphasis on their contributions towards precision nanomedicine.

## 1. Introduction

Nanotechnology has revolutionized modern oncology by providing the opportunity for therapeutic and diagnostic interventions at a nanoscale level, where materials display different physiological chemical behaviors than their bulk counterparts. Nanomaterials with sizes ranging from 1–100 nm are higher in surface area/volume ratio, and evince distinguishable changes in optical and electronic properties, as well as better interactions with cells, making them more attractive in biomedical applications. The shortcomings of traditional approaches gave rise to the development of several nano-systems, including liposomes, polymeric NPs, dendrimers, quantum dots and metal NPs, that aim to solve the issues associated with conventional chemotherapy such as poor solubility of drugs in water, nonspecific (systemic) toxicity across tissues (off-target effect), fast clearance from circulation and ineffectual targeting [1]. The targeting of tumors by utilizing the enhanced permeation and retention (EPR) effect and overcoming non-specific accumulation is a defining feature of nanomedicine, making it a landmark treatment that can overcome issues concerning abnormal vasculature and diseased lymphatic drainage. Moreover, alongside passive targeting, surface modification permits active targeting for cell receptors on the tumors, thus improving treatment specificity [2]. Nanomaterials are considered some of the most promising classes of nanocarriers, and metallic nanoparticles, especially gold nanoparticles, have been shown to be accidentally multifunctional agents with imaging ability, targeting ability, and drug delivery capability as well as capacities in radio sensitization and photothermics. This versatility, combined with their excellent biocompatibility characteristics, has made them the prime candidate for translational investigations in nanomedicine [3].

Gold nanoparticles have some intrinsic properties which render them extremely well-suited for oncological applications. The higher atomic number of gold (Z = 79), which leads to photoelectric absorption with the deposit of radiation doses, gives AuNPs the nature of a radiosensitizer under clinical practice when used in irradiative therapies. Gold exhibits a localized surface plasmon resonance (SPR) at the nanoscale, where conduction electrons oscillate in collective motion upon interaction with electromagnetic radiation. This property allows for efficient photothermal conversion under near-infrared (NIR) light to induce tumor-selective hyperthermia, and uses light-absorbing nanomaterials to deliver localized heat to impart cytotoxicity on cancer cells [4].

AuNPs are also chemically inert and oxidation resistant and they exhibit excellent affinity with thiol and amine groups, which allows them to bioconjugate with various therapeutic agents, targeting ligands, nucleic acids and polymers. Controlled size and morphology in fabrication enables charge optimization, externalizing activation, optimal pharmacokinetics, and optimized tumor penetration. The current mechanistically rational composite and complex therapy availability, together with well-established feasibility, identifies AuNP-based means as a unique addition to provisional diagnostic and therapeutic treatments in prostate cancer determined by powerful, defined molecular targets (e.g., prostatic specific antigen (PSA), prostatic acid phosphatase) and clinical radiogenic dependence [5].

This review aims to summarize and provide a reference for the literature surrounding gold nanoparticles in prostate cancer therapeutics development. This review therefore aims to summarize the clinical and molecular bases of prostate cancer and describe the physicochemical features and synthetic methods of AuNPs, their antitumor mechanisms and diagnostic and theranostics applications, and the preclinical evidence and clinical data, as well as dealing with toxicity issues along with regulatory aspects [6]. The review provides an overview of current knowledge in the field and identifies gaps in research to delineate routes forward for therapeutics development with AuNP specifically for the treatment of prostate cancer.

However, challenges such as tumor heterogeneity, treatment resistance, off-target toxicity and disease recurrence still pose difficulties in the clinical management of prostate cancer, even with the remarkable advances made by nanomedicine. Due to its unique physicochemical properties, such as adjustable size and morphology, localized surface plasmon resonance (LSPR), good colloidal stability, high ratio of surface-to-volume, easy surface modification, and biocompatibility, among many nanomaterials, gold nanoparticle (AuNP) has become one of the most studied nanoplatforms. These unique properties allow AuNPs to be used as multifunctional platforms for targeted drug delivery, molecular imaging, biosensing, photothermal therapy, radiosensitization, gene delivery and theragnostic applications in oncology. Additionally, in recent years the landscape of AuNP-based nanomedicine has been greatly expanded through multiple advances in precision oncology, ligand-mediated targeting, immunomodulation, multimodal imaging and combination therapies. Therefore, it would be important to provide a disease-specific review that also considers these new developments.

The current review presents novel findings on multi-modal therapeutic applications of gold nanoparticle-based strategies in prostate cancer from the recent literature that differ from previous reports. This review summarizes prostate cancer on a molecular basis as well as critically examining how the properties of nanoparticles such as size, morphology and surface chemistry affect its biological behavior, cellular uptake biodistribution and therapeutic performance. In addition, it describes surface engineering strategies, ligand-mediated active targeting, PSMA-directed nanoplatforms, stimuli-responsive drug delivery systems and intracellular trafficking mechanisms involving cisplatin-based conjugate for controlled drug release.

### Comparison of Gold Nanoparticles and Other Metallic Nanoparticles

Due to their unique physicochemical properties and multifunctional therapeutic capabilities, metallic nanoparticles have garnered significant attention in oncology. They include gold, silver, iron oxide, platinum, copper oxide and silica-coated metallic nanoparticles, which are particularly promising for cancer diagnosis and therapy. However, all types of metallic nanoplatforms have their own specific biological modalities that affect their use in biomedicine.

Silver nanoparticles (AgNPs) show strong antimicrobial effects and inherent cytotoxicity against cancer cells by means of intracellular accumulation of reactive oxygen species (ROS)-related oxidative stress. Nevertheless, their clinical translation has been hampered by the relatively high cytotoxicity towards healthy tissues, poor long-term biocompatibility and potential systemic toxicity after multiple cycles of administration. In contrast, iron oxide-based nanoparticles exhibit advantageous magnetic properties which lead to potential applications in MRI, magnetic hyperthermia and magnetically mediated drug delivery. Iron oxide nanoparticles mostly lack the characteristic optical properties for photo-thermal treatment and photo-acoustic imaging.

Platinum nanoparticles hold promise for catalytic activity and radiosensitization properties, short-listing them as possible alternatives to platinum-based chemotherapeutics. However, their usage in biomedicine is still limited by concerns for oxidative stress, nephrotoxicity and low surface functionalization. In particular, copper-based nanoparticles have recently been investigated as photothermal agents due to their strong near-infrared absorbance coupled with lower production costs. In contrast, copper nanomaterials exhibit poorer chemical stability and stronger oxidation susceptibility than gold nanostructures.

Gold nanoparticles (AuNP) have many unique characteristics that make them an attractive option for prostate cancer nanomedicine among the inorganic nanomaterials currently available. Gold has superb chemical inertness, impressive resistance to oxidation, relatively low intrinsic toxicity and high biocompatibility. Even more exciting, AuNPs have tunable localized surface plasmon resonance (LSPR) that efficiently absorbs light and converts it into heat through photothermal treatment upon near-infrared (NIR) irradiation. This technology underlies a variety of biomedical applications, such as photothermal therapy, surface-enhanced Raman scattering (SERS), photoacoustic imaging and multimodal theranostics.

Another distinct advantage of AuNPs is their remarkable surface chemistry. The drug delivery system, modified with thiol, works by utilizing the strong gold–thiol interactions for stable conjugation of antibodies, peptides, aptamers, nucleic acids, polymers and chemotherapeutics, enabling highly specific targeted nanoplatforms. Surface functionalization allows for the addition of polyethylene glycol (PEG), prostate-specific membrane antigen (PSMA)-targeting ligands, folate, transferrin and other biomolecules that greatly enhance tumor-specific accumulation while decreasing nonspecific uptake by nontumor tissue.

Additionally, gold has a high atomic number (Z = 79), which affords excellent X-ray attenuation, and this leads to AuNPs being not only an effective radiosensitizer but also biomarkers for computed tomography (CT) contrast agents. Meanwhile, their nanoscale sizes, together with adjustable morphology, allow for the optimization of circulation time, tumor penetration, cellular uptake and intracellular drug release. All these attributes make AuNPs one of the most versatile inorganic nanoplatforms in the field of precision oncology.

However, several hurdles must still be overcome before clinical adoption at scale is possible. The primary hurdles, associated with organ accumulation, poor biodegradation bioavailability or high protein corona variability, large-scale manufacturing capability and lack of standardization by international regulatory bodies, can be addressed through extensive studies. Thus, the subsequent research must include the connection of bioactive and biodegradable gold nanostructures and better pharmacokinetic behavior and clearance profiles, which will lead to designed clinical studies and a more truthful translation into normal scientific practice.

## 2. Prostate Cancer: A Clinical and Molecular Overview

### 2.1. Epidemiology and Global Burden

Male prostate cancer is the most common form of the disease and second only to skin cancer among cancers diagnosed worldwide, leading to it account for almost 10% of all new cases of cancer, as well as being a major contributor to overall deaths from the disease. It is more prevalent in North America, Western Europe and Australia; but lower rates can be found in certain regions of Asia. Such variation reflects differences in genetic susceptibility, lifestyle risk factors and access to health care resources and screening practices. Broad implementation of prostate-specific antigen (PSA) screening has greatly improved early-stage diagnosis; however, it has also raised concerns, including overdiagnosis and overtreatment [7]. Age is the most significant risk factor; incidence surges after age 50. But while metastatic disease is a key therapeutic challenge, and the stage of PC where most prostate-related death occurs, here localized disease is typically curative.

### 2.2. Risk Factors and Pathogenesis

PC pathogenesis is multifactorial, with a complex interplay of genetic, hormonal, environmental and inflammatory factors. A family history and inherited mutations—especially in the BRCA1 and BRCA2 genes—greatly increase the risk. Androgens play a central role in driving disease initiation and progression, with testosterone being converted to dihydrotestosterone (DHT) via 5α-reductase, which binds to the androgen receptor (AR) and alters gene transcription related to cellular proliferation and survival [8]. Androgen receptor signalling dysregulation drives tumorigenesis and promotes therapy resistance. Several cytokines of the prostate microenvironment associated with chronic inflammation might also augment neoplastic transformation by multiple cytokine-mediated pathways of signaling. Common molecular alterations in prostate cancer include PTEN deletion, TMPRSS2–ERG gene fusion, p53 mutations and activation of survival signalling pathways. Elimination of the apoptotic process, uncontrolled proliferation and metastasis are driven by accumulative genetic and epigenetic alterations [9].

### 2.3. Molecular Pathways Involved in Prostate Cancer

**Figure 1:**
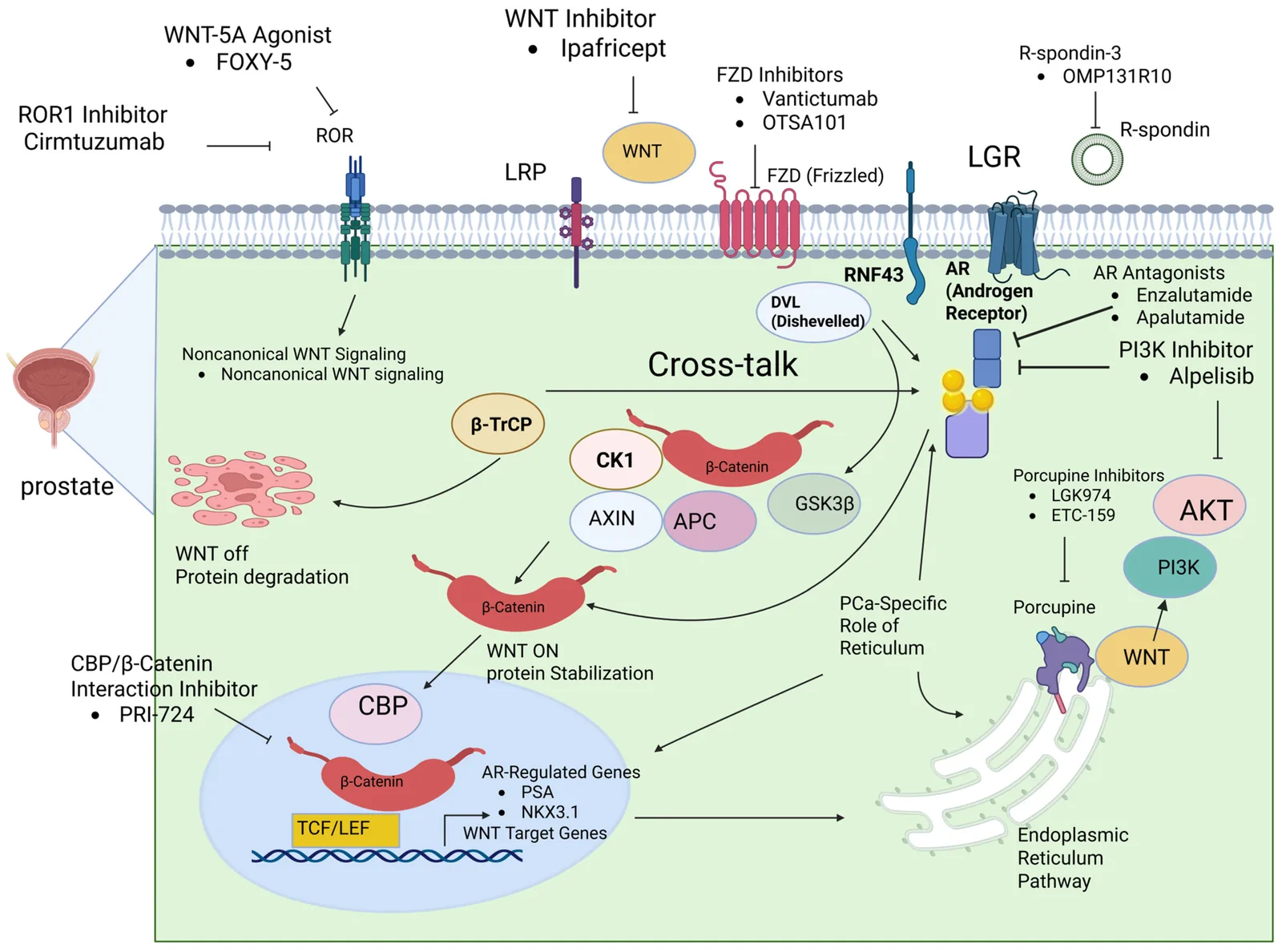
Prostate cancer signaling pathway and targeting therapeutic strategies. Created in BioRender. Hani, U. (2026) https://BioRender.com/3jeo9fq (accessed on 14 June 2026).

Several relevant oncogenic signalling pathways are involved in prostate carcinogenesis and progression. The PI3K/AKT/mTOR pathway is commonly activated, with PTEN loss or mutation occurring in many cancers, leading to enhanced survival characteristics and metabolic reprogramming. PI3K/AKT signalling and androgen receptor pathways interact synergistically to promote resistance to androgen deprivation therapy [10]. Aberrant Wnt/β–catenin signalling promotes tumor invasiveness, while proliferation and differentiation are driven by MAPK signalling pathways. Binding of TNF to its receptors activates the NF-κB (nuclear factor kappa-light-chain-enhancer of activated B cells)-dependent inflammatory signalling pathway, promoting the production of cytokines/chemokines, angiogenesis and resistance to apoptosis (Figure 1). These molecular pathways converge to allow for tumorigenesis, metastatic potential and therapy resistance, emphasizing the need for multimodal therapeutic strategies that can act on target and the ability to simultaneously hit multiple axes—an arena where nanotechnology-based therapeutics provide distinctive advantages [11].

The interaction network between all these molecular players is summarized in the diagram above, where different therapeutic targets (arrows) can be observed. WNT secretion begins at the level of the endoplasmic reticulum, where WNT ligands are acylated by Porcupine 17, an important step that can be inhibited with small molecules such as LGK974 or ETC-159. WNT ligands are secreted and interact with the cell membrane Frizzled (FZD) and LRP receptor complexes. This interaction is a critical therapeutic target, and the FZD solubility traps, such as WNT-trap Ipafricept and other multiple FZD inhibitors (e.g., Vantictumab), are in clinical studies aiming to hinder this binding [12]. In addition, the pathway signaling is regulated by coreceptors (e.g., ROR, Cirmtuzumab target) and the R-spondin/LGR axis that controls membrane receptor stability.

In the absence of WNT signaling (“WNT off”), cytoplasmic destruction complex components AXIN, APC, GSK3/beta and CK1 phosphorylate \beta-catenin function for ubiquitination by beta-TrCP prior to proteasomal degradation. In contrast, in a “WNT-on” condition, where the WNT pathway is activated and dishevelled (DVL) proteins accumulate, the destruction complex will be released to allow for the accumulation of β-catenin proteins. This allows \beta-catenin to translocate to the nucleus. Therefore, the mechanism-blocking cross-talk between stabilized \beta-catenin, and androgen-receptor (AR) and the PI3K/AKT pathway is pivotal in prostate cancer. AR antagonists, for example, enzalutamide, or PI3K inhibitors like alpelisib are typically used to counteract these interactions [13]. β-catenin subsequently acts in the nucleus as a co-activator of TCF/LEF transcription factors. This recruitment is mediated by the co-activator CBP, and such interaction can be blocked by PRI-724. The transcriptional complex thus formed promotes expression of WNT target genes and prostate-specific markers such as PSA and NKX3, which ultimately leads to increased survival, growth and induction of CRPC [14].

### 2.4. Current Diagnostic and Therapeutic Approaches

Follow-up for PC depends on the severity of the disease, as well as individual comorbidity and molecular characteristics. The localized disease is often treated with radical prostatectomy or radiotherapy. While they are effective in the primary stages, recurrence is possible and further treatment may still be needed [15]. ADT has long been a cornerstone of treatment for advanced disease, which is usually treated with hormonal therapy. In reality, potent antiproliferative agents on prostate cancer cells (such as luteinizing hormone–releasing hormone [LHRH] agonists and antagonists and post-luteal inhibitors, androgen receptor and CYP17 inhibition) reduce androgen signalling but are ultimately responsible for the emergence of castration-resistant disease. In metastatic environments chemotherapeutic agents (especially docetaxel and cabazitaxel) are employed; nevertheless, they impose systemic toxicity. Results from targeted therapy, including the use of PARP inhibitors in BRCA-mutated tumors, have seen improvements in outcomes for certain patient subgroups. Notwithstanding these advances, major limitations remain, such as drug resistance, toxicity, incomplete tumor killing and lack of specificity [16].

#### 2.4.1. Surgery and Radiotherapy

The cornerstone treatments for localized and locally advanced prostate cancer are radical prostatectomy and radiotherapy. This surgery removes the entire prostate gland and some surrounding tissue, including the seminal vesicles, though it may or may not include pelvic lymph node dissection. There have been recent advances concerning nerve-sparing approaches, in addition to robotic-assisted laparoscopic techniques that provide a marked advantage regarding functional outcomes, especially with respect to extra-urethral continence and potency [17]. One major clinical burden still, which is also relevant to patients with higher urine–pathological features, is constituted by postoperative complication disturbances as well as biochemical recurrences. EBRT or brachytherapy can be performed if less aggressive measures have failed. Various radiotherapy modalities such as intensity-modulated radiation (IMRT) and image-guided radiotherapy (IGRT), as well as stereotactic body radiotherapy (SBRT), have shown that these techniques can deliver the planned dose to the prostate gland while protecting surrounding normal tissues from being killed, including rectum and bladder. Despite such advancements, radiation toxicity and local recurrence as well as emergence of radioresistant tumor clones remain significant clinical issues. Radiation is the palliative to bone metastases in a metastatic setting [18]. Prostate tumors are heterogeneously radiosensitive, and AuNPs, readily available radiosensitizers, have been investigated to enhance therapeutic efficacy with minimum collateral damage.

#### 2.4.2. Hormonal Therapy

Hormonal therapy, particularly androgen deprivation therapy (ADT), is the cornerstone of systemic treatment in advanced and metastatic prostate cancer. The biological basis of this derives from prostatic epithelial cells being reliant upon androgens through numerous pathways, predominantly and notably, androgen receptor (AR) signalling. The execution of ADT can be either surgical (orchiectomy) or pharmacological, with the latter using luteinizing hormone-releasing hormone (LHRH) agonists or antagonists to decrease circulating levels of testosterone [19]. Androgens associate in the cytoplasm with their cognate receptor and signal through cross-talk with other intracellular signalling pathways, such as PI3K or MAPK, which can be blocked in resistant settings with ADT by antiandrogens such as enzalutamide and apalutamide, which directly interfere with ligand-binding or nuclear translocation. CYP17 inhibitors, including abiraterone, lower androgen production itself [20]. Although ADT offers an almost universal response in terms of tumor burden reduction and lessening of symptoms, this is invariably followed by the development of castration-resistant prostate cancer (CRPC), and a biological state that sees continued androgen receptor activity in the presence of significantly decreased levels of circulating androgens. Mechanisms of resistance include amplification of the AR gene, expression of splice variants that lack ligand-binding domains, intertumoral androgen biosynthesis and activation of alternative survival routes such as PI3K/AKT signalling. Androgen deprivation therapy is linked to metabolic syndrome, cardiovascular risk, osteoporosis and poor quality of life. Such limitations emphasize the pressing need for much-improved therapeutic approaches that can circumvent resistance mechanisms and augment tumor-specific cytotoxicity [21].

#### 2.4.3. Chemotherapy and Targeted Therapy

As for the role of chemotherapy in the treatment of metastatic and castration-resistant PC, at present, the mainstay of chemotherapeutic agents is docetaxel, a microtubule-stabilizing agent taxane that has demonstrated survival benefits in metastatic castration-resistant prostate cancer (mCRPC). It prevents microtubule depolymerization, leading to mitotic arrest and death. Cabazitaxel is a second-generation taxane employed when docetaxel resistance occurs, as it has less specificity for the drug efflux transporters [22]. Taxane-based therapy, however, is associated with substantial systemic toxicities, including myelosuppression, neuropathy and gastrointestinal conditions. In recent years, molecularly targeted medicines have emerged that have widened treatment possibilities. Approved poly (ADP–ribose) polymerase (PARP) inhibitors, represented by olaparib and rucaparib, function in individuals with abnormalities in homologous recombination repair genes such as BRCA1/2, not only in late-stage disease with radioligand therapy, but also using lutetium-177-labelled agents targeting prostate-specific membrane antigen (PSMA) and other components [23]. Despite these advances, treatment resistance, limited durability of response and off-target toxicity still predominate.

#### 2.4.4. Limitations of Conventional Therapies

Although conventional treatment methods have greatly improved survival rates, there are still significant disadvantages. For example, surgical intervention can result in functional morbidities (e.g., urinary incontinence and erectile dysfunction), so it may also impact long-term quality of life. Although precision is increasing in radiotherapy, this treatment modality remains potentially acutely and chronically genitourinary- and gastrointestinal-toxic, a fact that can lead to secondary malignancies. Most advanced cases eventually develop resistance to hormonal therapy and become aggressive, treatment-refractory, and castration-resistant disease [24]. Classical chemotherapeutic agents lack targeting capabilities and produce systemic toxicity, leading to undesirable side effects that limit their effective therapeutic use. However, tumor heterogeneity and activation of adaptive signalling pathways contribute to therapeutic resistance and disease progression. Micro-metastatic disease and circulating tumor cells escape standard treatment, leading to recurrence. These limitations spurred investigations into alternative modalities such as gold nanoparticle-based systems with favorable properties for the precise delivery of concurrent multimodal therapies, radio sensitization, and reduced off-target toxicity. Nanotechnology-based interventions could potentially overcome the natural limitations posed by current prostate cancer therapies through enhancement of therapeutic specificity and reduction of resistance pathway targeting [25].

## 3. Gold Nanoparticles (AuNPs): Fundamental Aspects

### 3.1. Historical Perspective and Development

The therapeutic utility of gold has a several-decades-long history; however, the advent of nanotechnology enabled the generation of gold at nanoscale, and this opened up new avenues for a broad spectrum of biomedical applications. Stable, monodisperse ultra-small gold nanoparticles were constructed using controlled colloidal synthesis, where citrate reduction and thiol-stabilization techniques enabled the formation of nanostructures. Subsequent work addressed this limitation, for example, via seed-mediated growth leading to substrates of anisotropic compositions such as nanorods and nanostars. These advancements have expanded the utility of AuNP in cancer owing to its tunable optical, electrical, and surface characteristics [26].

### 3.2. Gold Nanoparticle Platform Classifications

Gold nanoparticles (AuNPs) comprise a complex family of nanostructures that have different designs in terms of morphology, dimension, composition, and optical properties that strongly govern their biological behavior and therapeutic efficacy. Recent advances in nanofabrication methods have permitted the synthesis of different gold nanostructures with adjustable physicochemical properties useful for precision oncology. For prostate cancer, the appropriate AuNP platform is selected depending on their biomedical purpose (e.g., targeted drug delivery, molecular imaging agent, PTT and radiosensitizer agent, as well as biosensing or theragnostic integration).

#### 3.2.1. Gold Nanospheres

Gold nanospheres are the most widely studied AuNP platform, due to their easy synthesis, high colloidal stability and flexible surface chemistry. Citrate reduction methods are meanwhile the usual synthesis routes, and particles (5–100 nm in diameter) have been produced by these methods. Gold nanoparticles are characterized by a surface plasmon resonance peak close to 520 nm, making them useful for practical applications, colorimetric biosensing, molecular imaging and drug delivery.

Having a comparatively high surface-to-volume ratio aids in the conjugation of nucleic acids, peptides, antibodies and polymers via gold–thiol chemistry or electrostatic interactions. PEGylation of the surface importantly showed prolonged systemic circulation and reduced interaction with the opsonization of plasma proteins. In prostate cancer, nanospheres functionalized with PSMA-targeting ligands have shown significantly higher receptor-mediated cellular uptake and tumor accumulation and reduced off-target toxicity.

#### 3.2.2. Gold Nanorods

Gold nanorods exhibit anisotropic geometry within two spatial directions, the transverse and longitudinal axes that give rise to transverse and longitudinal plasmon resonance bands. Their longitudinal plasmon resonance can be adjusted to occur in the biological window where tissue is highly transparent to optical frequencies. Thus, gold nanorods are recognized as one of the most efficient nanodrugs for photothermal therapy.

When irradiated in the NIR region, collective oscillation of conduction-band electrons converts absorbed optical energy into localized heat through a non-radiative relaxation process. Localized hyperthermia causes protein denaturation, mitochondrial dysfunction, membrane disruption, DNA damage and apoptosis of cancer cells, with sparse injury to the most proximate nearby normal tissues. Apart from using photothermal therapy, gold nanorods also demonstrate enhanced photoacoustic imaging performances and have been successfully used as theragnostic items to achieve the integration of drug delivery, imaging and thermal ablation all at once.

#### 3.2.3. Gold Nanoshells

Gold nanoshells are composed of a dielectric core (usually silica) covered by a very thin gold shell. Their plasmon resonance can be tuned from the visible spectrum to the near-infrared region by just changing the relative size of core and shell, resulting in a strong optical absorption at depths of tissue. Such tunable optical characteristics of gold nanoshells render them highly promising candidates for non-invasive photothermal therapy.

In addition to thermal ablation, gold nanoshells offer large surface areas that can simultaneously incorporate chemotherapeutic drugs, imaging probes, targeting ligands and nucleic acids. The hollow architecture allows for a hierarchical therapeutic design that supports dynamic combined therapy (chemotherapy and photothermal) as well as molecular imaging, all within one nanoplatform.

#### 3.2.4. Gold Nanocages

Gold nanocages are porous hollow nanostructures typically obtained from galvanic replacement reactions with silver. Their hollow core endows them with an exceptionally high loading capacity for therapeutic molecules, and the porous wall allows the controlled and stimuli-responsive release of drugs.

Gold nanocages have not only exhibited strong absorbance in the near-infrared region but also very high photothermal conversion efficiency. Endogenous and exogenous stimuli can be used to trigger drug release: acidic pH, increased deprivation of glutathione concentration, reactive oxygen species in the tumor microenvironment, or laser irradiation. Thus, gold nanocages became potential candidates as a multifunctional nanocarrier for controlled drug delivery and multimodal cancer therapy.

#### 3.2.5. Gold Nanostars

Gold nanostars have many sharp spikes (protrusions) extending from their center and can exhibit several electromagnetic “hot spots” where the electric field is significantly enhanced. Such structural properties yield very intense surface-enhanced Raman scattering (SERS) signals, enabling gold nanostars to be excellent candidates for ultra-sensitive molecular detection and biosensing applications.

Branched morphology also contributes to photothermal conversion capability, as sharp tips facilitate concentration of electromagnetic energy after laser excitation. Gold nanostars are indeed ideal candidates for simultaneous SERS-guided diagnosis and imaging-guided photothermal therapy. Additionally, their large surface area favors efficient co-immobilization of antibodies, peptides and chemotherapeutic agents in achieving a targeted therapy against prostate cancer.

#### 3.2.6. Gold Nanoclusters

Gold nanoclusters are ultrasmall nanoparticles with diameters <3 nm. The differences in size and surface charge also exhibit quantum confinement effects, which leads to the result that nanoclusters have discrete electronic energy levels and fluorescence emission, while AuNPs are different from conventional nanosized particles with a classical surface plasmon resonance effect.

By virtue of their extremely small size, they possess partial renal clearance capability, which enables long-term organ accumulation to be reduced and biosafety to be improved compared with larger AuNPs. Fluorescent gold nanoclusters have shown excellent promise for high-resolution tumor imaging, intracellular tracing, biosensing, and targeted drug delivery. Although poor photothermal performance limits their use, their favorable pharmacokinetic profile directed them toward clinical translation.

### 3.3. Physicochemical Properties of AuNPs

Nanoparticles of gold have specific physicochemical properties different from bulk gold particles, which makes them very promising candidates for biomedical application. At the nanoscale, gold exhibits extremely strong surface effects: localized SPR, increased atomic number effects and high surface reactivity [27]. However, the size, shape and surface chemistry of nanoparticles can potentially optimize these properties for their uptake by cells, biodistribution and therapeutic effects.

#### 3.3.1. Size, Shape and Surface Charge

AuNPs’ biological behavior is highly size- and shape-dependent. Typically, for systemic circulation and tumor targeting, 10–50 nm nanoparticles are preferred due to the enhanced permeation retention (EPR) effect. Particles of ≤10 nm size undergo rapid renal clearance. Nanoparticles with diameters greater than 100 nm are mostly captured by the reticuloendothelial system [28]. Ironically, in studies of PSMA-targeted AuNPs of various dimensions (2 nm, 5 nm, 19 nm), the studies have shown the smallest possible diameter (2 nm) was most effectively absorbed by tumors in vivo and produced highest radiation dose enhancement, while larger particles exhibited more cellular internalization capability in vitro; therefore, size is of paramount significance to transport as well as interaction behavior during the ionizing radiation exposure determining therapeutic effect. Surface charge significantly impacts colloidal stability and non-specific interactions, fundamentally changing cellular uptake [28] and more radical kinetics (neutral or weak anionic surface chemistries—most commonly created with PEGylation) provide stealth and a longer circulation half-life [29].

The current evidence indicates that AuNPs with diameters of 20–80 nm offer the best compromise between long blood circulation, effective tumor penetration, and receptor-mediated endocytosis. Nanoparticles of this size preferentially accumulate in tumor tissue via the enhanced permeability and retention (EPR) effect, in which the leaky tumor vasculature and hindered lymphatic drainage enable passive nanoparticle entrapment. Additionally, nanoparticles with diameters around 40–60 nm frequently exhibit the highest rates of clathrin-mediated cellular uptake in prostate cancer cells, translating to improved intracellular drug delivery and efficacy.

In addition to particle size, nanoparticle morphology is a major contributor to cellular interactions, biodistribution, circulation behavior, and therapeutic effectiveness. Various gold nanostructures, such as gold nanospheres, nanorods, nanoshells, nanocages and nanostars, have different surface areas (SA), aspect ratios (AR) and optoelectronic characteristics that dictate their use in biomedicine. Gold nanospheres are still some of the most widely used nanoplatforms due to their relatively simple synthesis, high colloidal stability, and receptor-mediated cellular uptake uniformity. Gold nanorods, on the other hand, possess anisotropic geometry with minimally variable longitudinal plasmonic resonance in the near-infrared region, which makes them ideally suited for photothermal therapy. Their elongated shape adds extra surface area for interaction with cellular membranes and this leads to a higher photothermal conversion efficiency compared to spherical nanoparticles.

Morphology also affects circulation behavior; Usually, the rod-like nanoparticles demonstrate more systemic circulation time than their sphere-like counterparts, since they are less likely to be taken away by phagocytic uptake; moreover, larger specific surface areas of highly branched structures facilitate conjugation with either targeting ligands or imaging probes. Therefore, adjusting the geometry of nanoparticles permits us to customize AuNPs for particular diagnostic and therapeutic uses.

Surface charge is of paramount importance in nanoparticle–cell interaction. Positively charged AuNPs are more effective in cellular internalization since electrostatic attraction between cationic nanoparticles and negatively charged phospholipid membranes help. But a positive charge also induces hemolysis, complement activation and non-specific interactions with healthy tissues.

Negatively charged and near-neutral nanoparticles typically show better colloidal stability and lower systemic toxicity. PEG (polyethylene glycol) is still the most used strategy for modifying surfaces and minimizing protein adsorption, increasing blood half-life, reducing macrophage uptake and ultimately enhancing passive tumor accumulation. PEGylation also prevents aggregation under physiologic conditions and increases nanoparticle circulation stability.

#### 3.3.2. Localized Surface Plasmon Resonance (LSPR)

Localized surface plasmon resonance (LSPR) is now one of the so-called general physicochemical properties unique to gold nanoparticles. The LSPR is the collective oscillation of conduction-band electrons induced by the coherent interaction of AuNPs with electromagnetic radiation of a proper wavelength. This phenomenon leads to ultra-strong optical absorption and scattering capabilities that most inorganic nanomaterials lack [30].

The various factors on which the wavelength of plasmon resonance depends are particle size and morphology, along with surrounding dielectric environment, and also interparticle spacing. In contrast, the surface plasmon resonance of gold nanospheres is usually observed at 520 nm while anisotropic structures, including nanorods, nanoshells and nanostars, can be optimized to absorb in the near infrared (NIR) biological window (700–900 nm), a spectral region characterized by minimal absorption in tissue and maximal light penetration.

After laser irradiation, hot electrons are rapidly lost non-radiatively via electron- or phonon-scattering processes and thermalization of absorbed optical energy into localized heat. This photothermal conversion is the principle of AuNP-mediated photothermal therapy, allowing selective killing of tumor tissue and leaving adjacent healthy structures unharmed.

#### 3.3.3. Effects on Cellular Uptake and Trafficking of Molecules

After accumulating in the tumor environment, AuNPs bind to the cellular membranes by receptor-mediated or non-specific endocytosis. Depending on the nanoparticle size, surface chemistry and cell type, the major cellular internalization pathways are primarily clathrin-mediated endocytosis, caveolae-mediated endocytosis macropinocytosis and phagocytosis.

Ligand-functionalized AuNPs bind to prostate-specific membrane antigen (PSMA), transferrin receptors, folate receptors or integrins, and provide a receptor-mediated uptake of the conjugated cells and enhance drug delivery into the cells. Following internalization, nanoparticles undergo trafficking through early endosomes and late endosomes, and finally to lysosomes. To achieve effective nucleic acid delivery or other type of gene therapy, successful escape from the endosomal compartment is critical to prevent lysosome degradation and allow therapeutic cargo to access sites of action in either the cytoplasm or nucleus.

#### 3.3.4. Influence of Biodistribution or Therapeutic Efficacy

The in vivo biodistribution and therapeutic performance of AuNPs are ultimately determined by the balance between the effects of NP size, morphology, surface chemistry, and LSPR. Controlled drug release, prolonged systemic circulation, increased uptake by tumor tissues and cells and improved therapeutic index can be realized through engineering of nanoparticles. Recent progress has led to the development of novel multifunctional AuNPs, which combined with their optimized physicochemical properties, have important functions such as active targeting, imaging, controlled drug-releasing performance, photothermal conversion and radiosensitization. These next-generation nanoplatforms have the potential to significantly enhance prostate cancer diagnosis and treatment, while at the same time enabling further clinical translation in this field.

### 3.4. Synthesis Methods

#### 3.4.1. Chemical Reduction Methods

The method most commonly used to synthesize AuNPs is chemical reduction. Gold salts in aqueous systems are chloroauric acid reduced via sodium citrate, sodium borohydride or ascorbic acid. Citrate reduction is a widely used standardized method for preparing relatively monodisperse gold nanoparticles with ~ spherical structures and tunable size controlled by the precursor concentration and variation in temperatures [31]. Stronger reducing agents like sodium borohydride produce smaller nanoparticles with much narrower size distributions. These organic-phase synthesis methods, which lead to thiol-capped AuNPs, are vital for the surface stability of a well-defined structure and initiate ligand-exchange chemical reactions. However, chemical reduction methods are reproducible and scalable, and residual chemicals and surfactants typically require extensive removal to ensure biocompatibility.

#### 3.4.2. Green Synthesis Approaches

The green synthesis of AuNPs can be a sustainable and environmentally friendly approach to chemical reduction. Such processes with biological materials (vegetable extracts, microorganisms and polysaccharides), ones that follow the principles of green chemistry as reducing and stabilizing agents and limit toxic wastes while decreasing energy utilization and increasing their biocompatibility would be important in cancer nanomedicine. Chloroauric acid (HAuCl_4_) is used to reduce gold from aquatic solutions in regular production; phyto-chemicals such as flavonoids and phenolics are exploited where there is a visible color change due to SPR [32]. This technique yielded AuNPs within the size range of 10–50 nm, which indicated UV–Vis peaks between 520 and 540 nm for effective NP biosynthesis. For instance, AuNPs (18 ± 4 nm) prepared through leaf extract-mediated methods exhibited excellent colloidal stability and cytocompatibility (>85% survivability in normal fibroblast cells with their respective doses). The findings describe the microbial synthesis by which AuNPs are generated from 5–40 nm using bacterium fungus and algae based on different parameters of optimum pH, temperature, precursor concentration etc. [33]. The extracellular fungal production method has been used for the synthesis of monodispersed (~12 nm) and crystallized AuNPs. Biological syntheses of AuNPs exhibit comparable photothermal conversion efficiencies (30–40%) under NIR irradiation to chemically prepared analogs. Moreover, polysaccharides and protein-assisted synthesis offer greener alternatives where natural polymers help in the reduction and stabilization of AuNPs that are more interactive with cancer cells, compared to the corresponding moieties that exhibit higher absorption efficiencies in vitro [34]. Chitosan-coated AuNPs show 1.5–2-fold higher internalization than citrate-stabilized AuNPs.

#### 3.4.3. Seed-Mediated Growth Techniques

**Figure 2:**
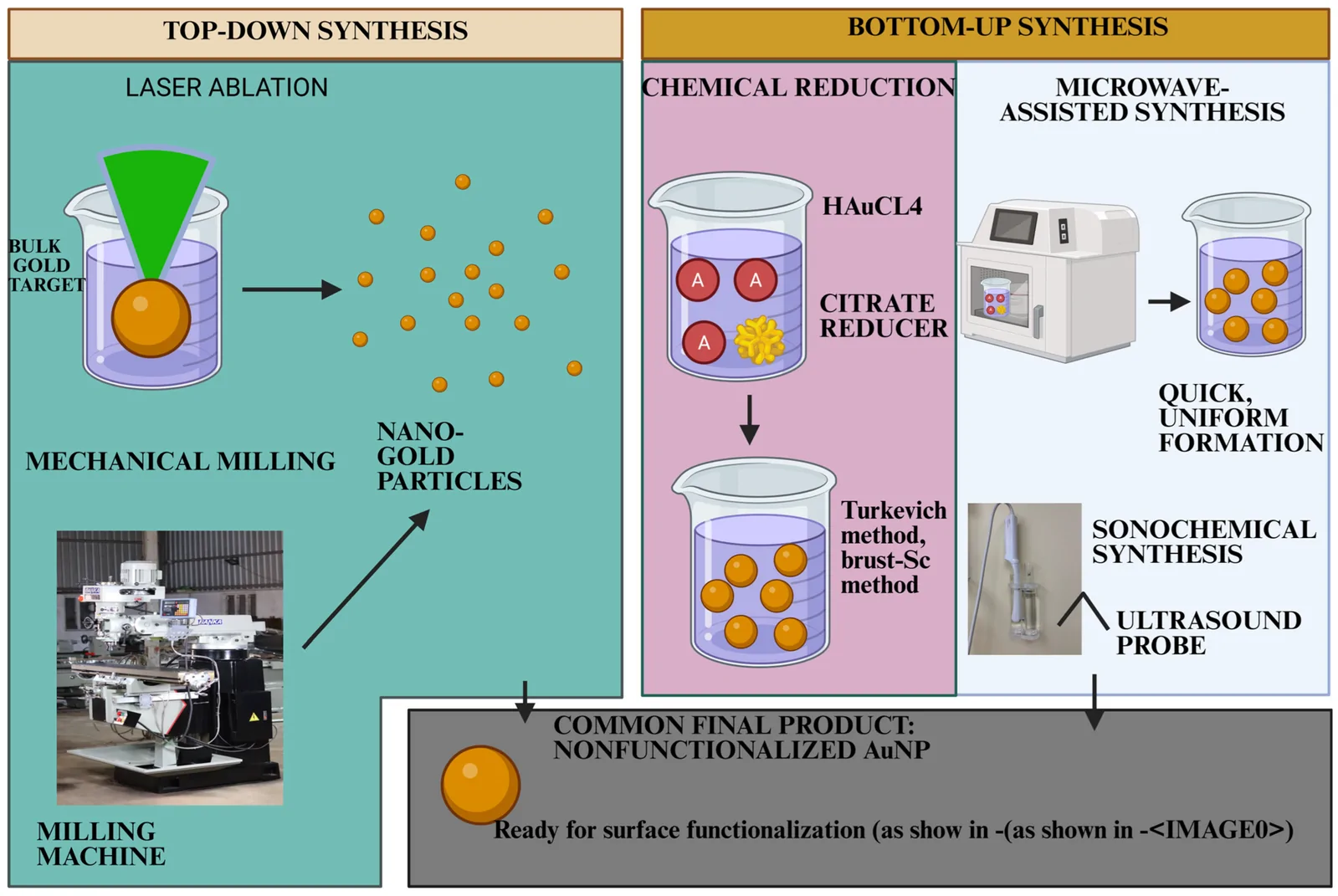
Synthesis method of gold nanoparticle. Created in BioRender. Hani, U. (2026) https://BioRender.com/ys06zzy (accessed on 14 June 2026).

Seed-mediated growth is a sophisticated and reliant method for preparing anisotropic AuNPs, including the wholesale use of AuNRs, nanostars and nanoplates, with good control of size and morphology. The process comprises of two steps: (i) the reduction of HAuCl_4_ into metallic gold in an aggressive chemical reaction for generation of small colloidal gold seeds (usually 2–5 nm), e.g., with sodium borohydride, and (ii) the growth of these seeds into bigger particles in a second solution still comprising HAuCl_4_ but at a lower concentration, soluble reducing agents ensuring gentler conditions than during seed formation [e.g., ascorbic acid] and face-template surfactants like CTAB [35]. This strategy enabled us to control the morphology in a selective fashion by tailoring multiple reaction parameters: seed concentration, silver ion amount, surfactant ratio, and temperature. Typical dimensions of the gold nanorods made by the seed-mediated method will be around 40 to 80 nm in length, and have a width of 10 to 20 nm and an aspect ratio of about 3–5 (Figure 2). A UV–visible study reveals the existence of two plasmonic peaks, one is a transverse peak around 520 nm and another is a longitudinal tunable peak from 650 to 900 nm, rendering this system applicable for near-infrared (NIR) photothermal therapy [36]. For near-infrared irradiation, previously published data on seed-grown nanorods suggest photothermal conversion efficiencies in the range of 35–50%, [36] which still suggests their utility for tumor ablation [37]. Disadvantages of the material that have been found to date are CTAB-related cytotoxicity, a need for surface modification (e.g., PEGylation) to improve prospective biocompatibility, and in vivo lifetime, though the products exhibit remarkable shape control and optical tunability. This means that seed-mediated growth is a reproducible and robust strategy to generate architecturally relevant AuNPs for oncological applications.

### 3.5. Characterization Techniques

Characterization of AuNPs involves thorough analysis of their physicochemical, structural, optical and biological features as potential agents for treatment to establish safety before use in therapeutic applications against prostate cancer. Morphological characterization is mainly carried out by transmission electron microscopy (TEM) and scanning electron microscopy (SEM), which gain clear information about the size, shape, and dispersion of particles associated with three-dimensional surface profile obtained from the atomic force microscopy (AFM) [38]. Hydrodynamic diameter and polydispersity index are measured by dynamic light scattering (DLS) analysis, and zeta potential measurements provide additional information about surface charge (surface charge can influence bioavailability in vivo) and governed colloidal stability, which is predictive of behavior in vivo [39,40,41].

UV–visible spectroscopy data indicates that the particles are formed by using significant aspects of classical surface plasmon resonance (SPR); a peak was detected, whereas Fourier transform infrared spectroscopy (FTIR) analysis validates different functional groups and also their surface functions. X-ray diffraction (XRD) and selected area electron diffraction (SAED) are used to examine the structural and crystalline properties of as-prepared materials, while energy dispersive X-ray spectroscopy (EDS), X-ray photoelectron spectroscopy (XPS) and inductively coupled plasma mass spectrometry (ICP-MS) are employed to ascertain elemental composition and surface chemistry [42,43,44]. Thermal stability and ligand density grafted onto the nanorods may be determined with thermogravimetric analysis and differential scanning calorimetry. Additionally, drug-loading and release rates were assessed using high-performance liquid chromatography and/or spectrophotometric approaches [45,46,47]. The recommendations of the biological characterizations are important, since this data assures a rational design behavior for the future clinical translation of gold nanoparticle-based drugs for prostate cancer treatment [48,49].

## 4. Functionalization and Surface Engineering of AuNPs

### 4.1. PEGylation and Stability Enhancement

One of such extensively used surface modification processes for obtaining biocompatibility in nanoparticles and extending its circulation time in live organisms is polyethene glycol (PEG) conjugation. PEGylation reduces non-specific protein adsorption and recognition by the reticuloendothelial system (RES), thus increasing plasma half-life, while enhanced permeability and retention effect provides increased accumulation of drug in tumor tissues. As a result of applications in prostate cancer models, it was shown that compared to unmodified agents PEGylated AuNPs exhibit favorable biodistribution profiles including reduced hepatic and splenic uptake. Also, PEG shells afford steric stabilization against aggregation in physiomes free to conjugate targeting ligands or bioactive therapeutics. This demonstrates that in order to optimize stabilization efficiency and cellular uptake, careful characterization of PEG chain lengths and densities is crucial [50].

### 4.2. Ligands for Specificity (Antibodies, Peptides and Aptamers)

Targeting ligands (antibodies, peptides and aptamers) utilize AuNPs for PC therapy. Although plenty of literature data on ligand–drug and nanocarrier–disease interaction have been reported, the aspect which relevant to prostate cancer is highly investigational. Chemotherapy drugs conjugated on the surface of the nanoparticles provide active targeting against tumor-associated biomarkers and increase target-based accumulation at delivery through the target tumor site while also minimizing toxicity on healthy tissues [51]. Antibodies are a class of immunoglobulin molecules that can bind to and recognize certain antigens overexpressed on cancer cells. Monoclonal antibodies directed to these tumor-associated receptors that are responsive for prostate cancer can also be developed against prostate-specific membrane antigen (PSMA), epidermal growth factor receptor (EGFR), and others. In this manner, antibody-focused AuNPs may have make an increasing contribution to drug therapeutics. Nevertheless, the addition of antibodies could increase nanoparticle sizes, costs of fabrication and potential immunogenicity [52].

Peptides are relatively short chains (amino acid sequences) which can selectively ligate certain receptor/cellular markers. Compared with antibodies, these compounds have many advantages, such as small size, low immunogenicity, easy synthesis and better tissue penetration. As for targeted delivery, several MUC1-targeted agents (e.g., 4A, αv integrins) were designed to facilitate high tumor tissue accumulation of peptides with high-affinity binding [53]. Data could not be generated above a molecular weight of ~6000 Da such that sheer lower molecular weight yields a higher packing density of ligands on the nanoparticle surface, ultimately leading to optimal targeting efficacy. Aptamers are short single-stranded oligonucleotides (DNA or RNA) that can fold into unique three-dimensional structures and specifically bind to a broad range of molecular targets with high affinity. Aptamers, also known as “chemical antibodies”, have many benefits, including high stability, low immunogenicity and direct chemical modification, which allows for reproducible synthesis. The concept of aptamer-conjugated AuNP-based imaging as well as a localization-specific approach to targeting drug delivery can aid in the local deliverance of therapeutic agents to prostate cancer.

### 4.3. Active Targeting of GNPs in Prostate Cancer

The most prominent example of this is passive accumulation in tumor tissues via the enhanced permeability and retention (EPR) effect, which can improve drug localization by enhanced accumulation; however, EPR often results in rapid heterogeneous nanoparticle distribution, limiting therapeutic specificity. In order to bypass these limitations, strategies of active targeting have been created by taking advantage of the surface functionalization of AuNPs with ligands that may selectively target receptors overexpressed on prostate cancer cells. Active targeting can be accomplished by improving internalization of the drug–receptor complex into cells through receptor-mediated cellular uptake, thereby increasing intracellular concentrations, leading to improved therapeutic efficacy, while at the same time limiting side effects from non-specific uptake in non-target tissues.

Monoclonal antibodies are still one of the most commonly used targeting molecules in AuNP-mediated drug delivery. PSMA or epidermal growth factor receptor (EGFR)-specific antibodies have high binding affinity towards the specific ligand and can be used in selecting malignant cells as they exhibit specificity. The receptor binding subsequently leads to clathrin-mediated endocytosis, which facilitates the intracellular movement of chemotherapeutic agents, nucleic acids, or imaging probes through this nanoparticle–receptor complex. While antibody-mediated targeting has remarkable specificity, the clinical utility of antibodies is restricted by their relatively high molecular size and the complexity and cost of large-scale production.

Small molecule ligands such as folic acid and transferrin have also been frequently used for active targeting. Folate receptors are over-expressed in various malignant tumors and transferrin receptors have been found to be highly expressed on the surfaces of those cancer cells that rapidly proliferate because of their increased iron needs. Surface conjugation of AuNPs with folic acid or transferrin facilitates receptor-mediated endocytosis, enhancing intracellular drug uptake and therapeutic efficacy. Moreover, because of its affinity toward CD44 receptors expressed on cancer stem cells and metastatic tumor populations, hyaluronic acid has been also studied as a targeting ligand.

AuNPs are mainly internalized through clathrin-mediated or caveolae-mediated endocytosis after ligand–receptor interaction. Following internalization, nanoparticles are then recruited to early endosomes as well as late endosomes and subsequently released into lysosomal compartments. Efficient endosomal escape is important to avoid degradation of therapeutic cargo in drug and gene delivery applications. A number of approaches have been developed for inducing endosomal escape, including the use of pH, proton sponge materials, membrane-disruptive peptides and redox-sensitive linkers which ensure rapid delivery from within endosomes to the cytoplasm of drugs, siRNA, miRNA or plasmid DNA. These approaches greatly enhance intracellular availability and therapeutic efficacy.

### 4.4. Drug Delivery Attributes of Gold Nanoparticles: Loading, Release Kinetics and Intracellular Trafficking

#### 4.4.1. Drug Loading Capacity

A definite merit of AuNPs is that their tremendous drug-loading capability, which is associated with their size, topological aspects, surface chemistry, and functionalization strategy. Due to their large surface area, AuNPs can hold many therapeutic molecules through either physical adsorption, covalent conjugation, electrostatic interactions, or encapsulation within polymeric coatings. The most well-established of these approaches is covalent conjugation via gold–thiol (Au–S) chemistry, which allows the particle to be very stable in systemic circulation while preventing premature drug leakage.

AuNPs have been successfully conjugated with chemotherapeutic agents by means of thiol-containing linkers or polymeric spacers, including docetaxel, paclitaxel, doxorubicin and cisplatin. Besides these small-media drugs, AuNPs also have been confirmed to play a role in the delivery of nucleic acid therapeutics such as siRNA, miRNA, antisense oligonucleotides, plasmid DNA and CRISPR-associated gene-editing systems. These multiple therapeutic agents can be added at the same time to realize combination therapy, which allows a single nanoplatform for chemotherapy, gene therapy, and photothermal treatment.

#### 4.4.2. Controlled Drug Release Kinetics

In order to achieve tumor-targeted delivery for efficient cancer therapy, it is critical to achieve well-controlled drug release at the site of pathogenesis. The drug release from AuNPs is dependent on the strength of drug–nanoparticle interactions, linker chemistry and tumor microenvironment descriptions. As drugs are mainly contained by electrostatic or hydrophobic forces that are relatively weak, conventional adsorption-based systems generally show a rapid burst release. This is a rather simplistic strategy; however, premature release of the drug may diminish therapeutic efficacy while increasing systemic toxicity.

Many stimuli-responsive release systems have been devised to mitigate these restrictions. One of the most studied strategies is to invoke pH-responsive linkers, since the extracellular tumor microenvironment (pH 6.5–6.8) and intracellular endosomal compartments (pH 5.0–5.5) can be significantly more acidic than normal physiological conditions [1]. Using acid-labile linkages (e.g., acylhydrazone, imine or acetal), these formulations can be selectively cleaved to enable local drug release conditions in cancer tissues.

#### 4.4.3. Intracellular Trafficking of AuNPs

After the accumulation in tumor tissues, AuNPs need to be duly engulfed and transported into specific intracellular compartments for beneficial therapeutic effect. Most cellular uptake is mediated via receptor-mediated endocytosis, following interaction between targeting ligands and cell-surface receptors (e.g., PSMA, transferrin receptors, folate receptors or integrins). Due to differences in particle size, morphology, and surface properties, internalization may occur via clathrin-mediated endocytosis (CME), caveolae-mediated endocytosis (CavME), macropinocytosis or, rarely, phagocytosis.

Upon internalization, the nanoparticles are first found in early endosomes that progress to late endosomes and lysosomes [38]. Lysosomal degradation is a major bottleneck for intracellular drug and gene delivery as acidic lysosomal enzymes may degrade therapeutic cargo before it reaches the actual intracellular target. In this regard, the efficient endosomal escape is one of the most important determinants for successful AuNP mediated drug delivery.

Many engineering approaches have been explored to promote endosomal escape. Polyethyleneimine (PEI) and related proton sponge polymers can play a role in molecularly forced osmotic swelling of endosomes via accumulation of protons, leading to membrane rupture, resulting in delivery of the nanoparticles to the cytoplasm.

### 4.5. Drug Conjugation Strategies

Drug conjugation strategies, which have critical influence on drug loading capacity, stability, release kinetics and therapeutic effects, mainly determine the development and characteristics of the AuNP-based system for therapy toward prostate cancer. Which conjugation strategy it is that will be used will depend on the physicochemical characteristics of the drug, type of target release profile and biological application [54]. The most frequent approaches are based on physical adsorption, which involves attaching drug molecules to the surface of nanoparticle using weak interactions (e.g., electrostatic forces, hydrophobic interactions and van der Waals forces). It is a straightforward method that retains activity but could lead to early release from weak interactions.

This covalent modification results in a stable and strong bond between drug molecules and AuNPs via the formation of chemical bonds with some special groups on the drug compound itself as well as surface-engineered AuNPs. Because of their high affinity for gold, thiols (–SH) and amines (–NH_2_) used in thiol–gold chemistry are among the most adopted. In particular, EDC/NHS crosslinking agents provide a method to facilitate amide bonding of the carboxyl and amine groups [55]. They are considered to have stable circulation and facilitate drug release at varying physiological parameters. Cleavable linker strategies are well suited for applications of targeted cancer therapeutics. Moreover, the molecular synthesis of pH-sensitive linkers, redox-sensitive disulfide bond linkers, or enzyme-cleavable linkers also give rise to stimuli-responsive linkages which can facilitate site-specific drug release from nanocarriers under acidic conditions and high glutathione concentration in the tumor microenvironment [56].

Loading drugs could also lead to the coating or encapsulation in polymer layers (e.g., PEG, chitosan or PLGA) surrounding the gold core. This leads to steric stabilization, prolonged circulation time and controlled release of the drug. Besides chemotherapeutic drugs, nucleic acids (siRNA, miRNA, and plasmid DNA) may also be bound to AuNPs by electrostatic interaction or thiol-modified oligonucleotides for gene therapy [57]. Overall, these geometrically optimized schemes of drug conjugation will impart improved stability, tumor targeting and/or delivery efficiency, release kinetics and therapeutic potency in prostate cancer into nanoparticles.

### 4.6. Stimuli-Responsive Gold Nanoplatforms

Stimuli-responsive gold nanoplatforms represent a novel frontier in prostate cancer nanomedicine; they activate the release of therapeutic agents or function through one of numerous internal or external triggers. These smart systems would enhance treatment efficacy by reducing off-target toxicity and promote drug accumulation in tumors. Self-assessment systems (endogenous): During our work, the pH of the prostate tumor was slightly acidic and the ROS level was high; there was overexpression of other enzymes (e.g., HMGCR) as well, while at the same time intracellular GSH levels increased. Due to the specific pH-release property of gold nanoparticles, an acid–labile bond could be applied to release the chemotherapeutic drug in high-acid tumor tissues [58]. Among such designs are the redox-responsive systems, which have a disulfide bond that can be cleaved at high concentration of GSH to enable drug release intracellularly. Peptide linkers cleavable by specific tumor-associated enzymes enable selective activation and allow for the construction of an enzyme-sensitive scaffold.

External (exogenous) stimuli-responsive systems: These are triggered by an externally introduced physical stimuli including light, heat, ultrasound and radiation. However, the localized surface plasmon resonance (SPR) effect of gold nanoparticles increases their scattering cross-section, making them candidates whose photothermal therapy agents (PTT) have a very high potential. Upon irradiation of AuNPs with near-infrared (NIR) laser light, the energy is localized around the tumor tissue and converted to heat, causing selective destruction of tumor cells. Similarly, gold nanoplatforms act as radiosensitizers which augment the radiation-induced fragmentation of cancer cell’s DNA [59]. Externally regulated systems allow for site- and time-specific therapy delivery (Table 1).

**Figure 3:**
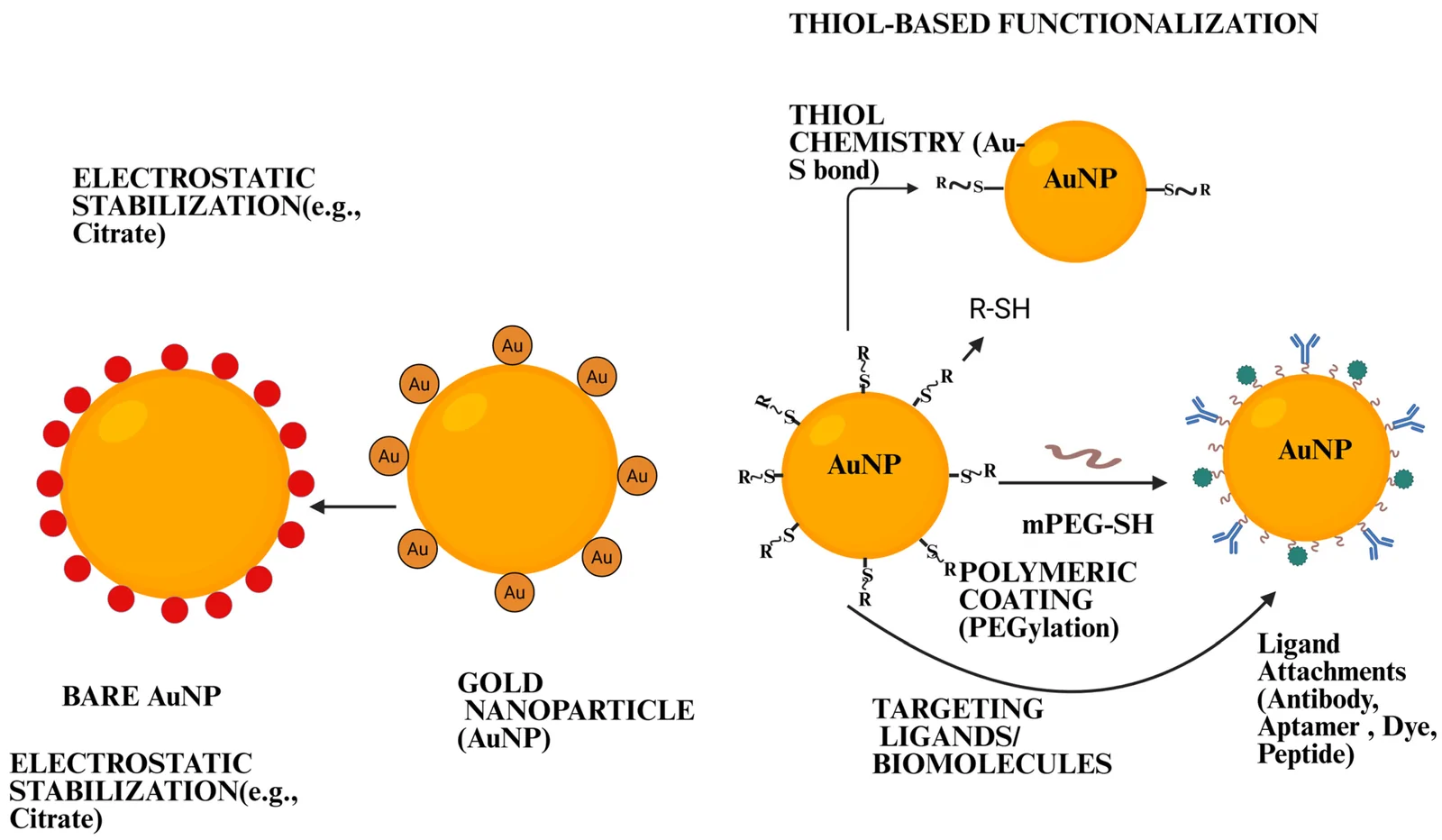
Surface functionalization strategies for gold nanoparticles. Created in BioRender. Hani, U. (2026) https://BioRender.com/e7uoe22 (accessed on 14 June 2026).

Dual or multi-responsiveness (e.g., pH- and photothermal responsive) DNM have great potential to get an additional stimulation to the right sites, enhancing therapeutic efficacy and specificity via drug release based on optimal triggering mechanism according to pathologists requirements. Such multimodal systems as this, implementing joint modalities (theranostics) as steps of the complete process, including chemotherapy, photothermal therapy and gene therapy, can play a role in any part of the entire process (Figure 3). Notably, the stimuli-responsive gold nanoplatforms also exhibit tunable tumor specificity and low systemic toxicity plus high therapeutic potency for controlled drug release behavior, indicating their promising utilizations in precision therapy of prostate cancer [60].

**Table 1:** Surface functionalization strategies for gold nanoparticles.

Surface Functionalization Strategy	Functional Molecule/Coating	Purpose of Modification	Key Research Findings	Application	Reference
PEGylation	Polyethylene glycol (PEG-SH)	Improve stability and circulation	PEGylation increased hydrodynamic diameter from ~65 to 70 nm, reduced zeta potential from +32 to +8 mV, and prolonged blood circulation by approximately 2-fold compared with uncoated AuNPs.	Drug delivery/imaging	[61]
Antibody conjugation	D2B monoclonal antibody	Target PSMA receptor	D2B-conjugated AuNPs exhibited approximately 3-fold greater cellular binding and significantly higher uptake in PSMA-positive prostate cancer cells than non-targeted nanoparticles.	Targeted therapy	[62]
Aptamer functionalization	PSMA aptamer	Target-specific recognition	PSMA aptamer-functionalized AuNPs detected PSMA with a limit of detection in the picogram mL^−1^ range and showed significantly enhanced uptake in PSMA-expressing cells.	Biosensing/targeted therapy	[63]
DNA conjugation	Thiolated DNA strands	Molecular recognition	DNA-functionalized AuNPs enabled sequence-specific hybridization with single-base mismatch discrimination for prostate cancer biomarker detection.	DNA biosensors	[64]
Polymer coating	Polyethyleneimine (PEI)	Gene delivery	PEI-coated AuNPs increased transfection efficiency by approximately 2–4-fold compared with naked DNA while maintaining >80% cell viability.	Gene therapy	[65]
Folate ligand conjugation	Folic acid	Target folate receptors	Folate-functionalized AuNPs demonstrated approximately 2-fold higher intracellular uptake in folate receptor-overexpressing cancer cells than non-targeted particles.	Targeted drug delivery	[66]
Transferrin conjugation	Transferrin protein	Target transferrin receptors	Transferrin-coated AuNPs significantly enhanced receptor-mediated endocytosis, resulting in approximately 2-fold greater intracellular accumulation.	Targeted delivery	[67]
Polydopamine coating	Polydopamine (PDA)	Enhance stability and biocompatibility	PDA coating increased particle diameter from ~65 to 88 nm, improved colloidal stability, and enhanced photothermal conversion efficiency under NIR irradiation.	Drug delivery	[68]
Protein coating	Bovine serum albumin (BSA)	Improve biocompatibility	BSA-coated Au nanoclusters maintained high colloidal stability in serum (>72 h) but exhibited limited renal clearance due to increased hydrodynamic size.	Imaging/therapy	[69]
Glutathione coating	Reduced glutathione (GSH)	Reduce toxicity	GSH-coated gold nanoclusters achieved approximately 36% renal clearance within 24 h, markedly reducing long-term organ accumulation.	Biomedical imaging	[70]
Lipid coating	Phospholipids	Improve membrane interaction	Lipid-coated AuNPs exhibited approximately 1.8-fold higher cellular uptake and improved serum stability compared with uncoated nanoparticles.	Drug delivery	[71]
Peptide functionalization	Tumor-targeting peptides	Improve tumor specificity	Peptide-functionalized AuNPs produced approximately 2–3-fold greater tumor accumulation than non-targeted nanoparticles in experimental tumor models.	Cancer targeting	[72]
Small molecule ligand coating	Citrate stabilization	Maintain nanoparticle dispersion	Citrate-capped AuNPs exhibited a zeta potential of approximately −35 to −40 mV, providing excellent colloidal stability and preventing aggregation.	Nanoparticle synthesis	[73]
Carbohydrate coating	Glycan ligands	Improve cell recognition	Glycan-functionalized AuNPs demonstrated significantly higher receptor-specific binding and improved discrimination between cancerous and normal cells.	Targeted diagnostics	[74]
Multifunctional surface modification	PEG + targeting ligand	Combined stability and targeting	Dual-functionalized AuNPs showed prolonged circulation, approximately 2-fold greater tumor accumulation, and improved therapeutic efficacy compared with single-functionalized nanoparticles.	Theranostics	[75]

## 5. Mechanisms of Action of Gold Nanoparticles in PC

The anticancer potentiation in PC has been shown previously through various synergistic mechanisms of action such as therapeutic agents and multifunctional delivery platforms. The mechanism of action is enhanced delivery of the drug. They enhance the solubility, stability and bioavailability of chemotherapeutic agents and promote their preferential aggregation into neoplasia through the EPR effect. When such nanoparticles are functionalized with one or more targeting ligands that bind to receptor lysines, for example, prostate-specific membrane antigen (PSMA), they actually undergo receptor-mediated endocytosis within the tumor and deliver their drugs in regions of cytotoxic activity [76,77].

AuNPs also function as radiosensitizers. However, since they have a high atomic number (Z = 79), these nanoparticles increase deposition of radiation dose in tumor tissue. Irradiation reactivates them, boosting secondary electrons and reactive oxygen species (ROS) that regulate DNA damage, cell cycle arrest and apoptosis. This mechanism augments the effect of radiotherapy, thus decreasing the radiation doses needed. Gold nanoparticles also induce cellular oxidative stress-mediated cytotoxicity, which involves an increase in intracellular ROS production, a disturbance of redox homeostasis triggering apoptosis via the intrinsic mechanism mediated by mitochondria in several cell types, resulting into severe mitochondrial damage [78]. They might alternatively regulate the signaling cascades responsible for cell proliferation and survival such as PI3K/Akt and MAPK pathways leading to suppression of tumorigenesis.

AuNPs are also being investigated in the applications of gene therapy and immunomodulation. They are used to deliver siRNA, miRNA or plasmid DNA for oncogene silencing or restore tumor suppressor gene function. Furthermore, they are likely to enhance antitumor immunity through antigen presentation or tumor-associated macrophage modulation mechanism. Overall, the progress of these multimodal platforms possessing unique drug delivery and photothermal ablation, or radiosensitization engendering oxidative pressure formation, or gene regulation has focused on gold nanoparticles acting as core center for research advancement in terms of prostate cancer therapeutic superiority in nanomedicine [79].

### 5.1. Pathways of Cellular Entry and Internalization

The therapeutic potency of AuNPs in the context of prostate cancer is contingent upon their effective internalization and an adequate intracellular delivery profile. One popular mechanism is known as clathrin-mediated endocytosis, which occurs with the binding of the nanoparticles to a specific cell surface receptor (e.g., PSMA for active targeting) internalized through a clathrin-coated vesicle [7]. As such, it is a great deal more specific than receptor-mediated uptake of ligand functionalized nanoparticles [80]. A second major route of endocytic entry comprises caveolae-mediated endocytosis (flask- shaped invaginations enriched in cholesterol and the protein caveolin). This route can also protect the nanoparticles from lysosomal degradation, which in turn leads to enhanced cytoplasmic delivery of sensitive therapeutic cargoes (siRNA or DNA).

Macropinocytosis is a nonspecific engulfment of fluid by means of the membrane ruffling and interaction with extracellular liquid. Indeed, this route allows larger and more dense nanoparticles to traverse cell membranes. Phagocytosis can also occur in immune cells such as tumor-associated macrophages in the tumor microenvironment. Overall uptake efficiency is primarily dictated by overall surface properties. The positive charge of AuNPs increases internalization and thus cytotoxicity due to the negative charge on the cell membrane [6]. The NP size range of 20–100 nm is important for uptake in tumor cells. For selective internalization, receptor–ligand-based interactions can be used for antibody, peptide or aptamer-based functionalized surfaces [81].

**Figure 4:**
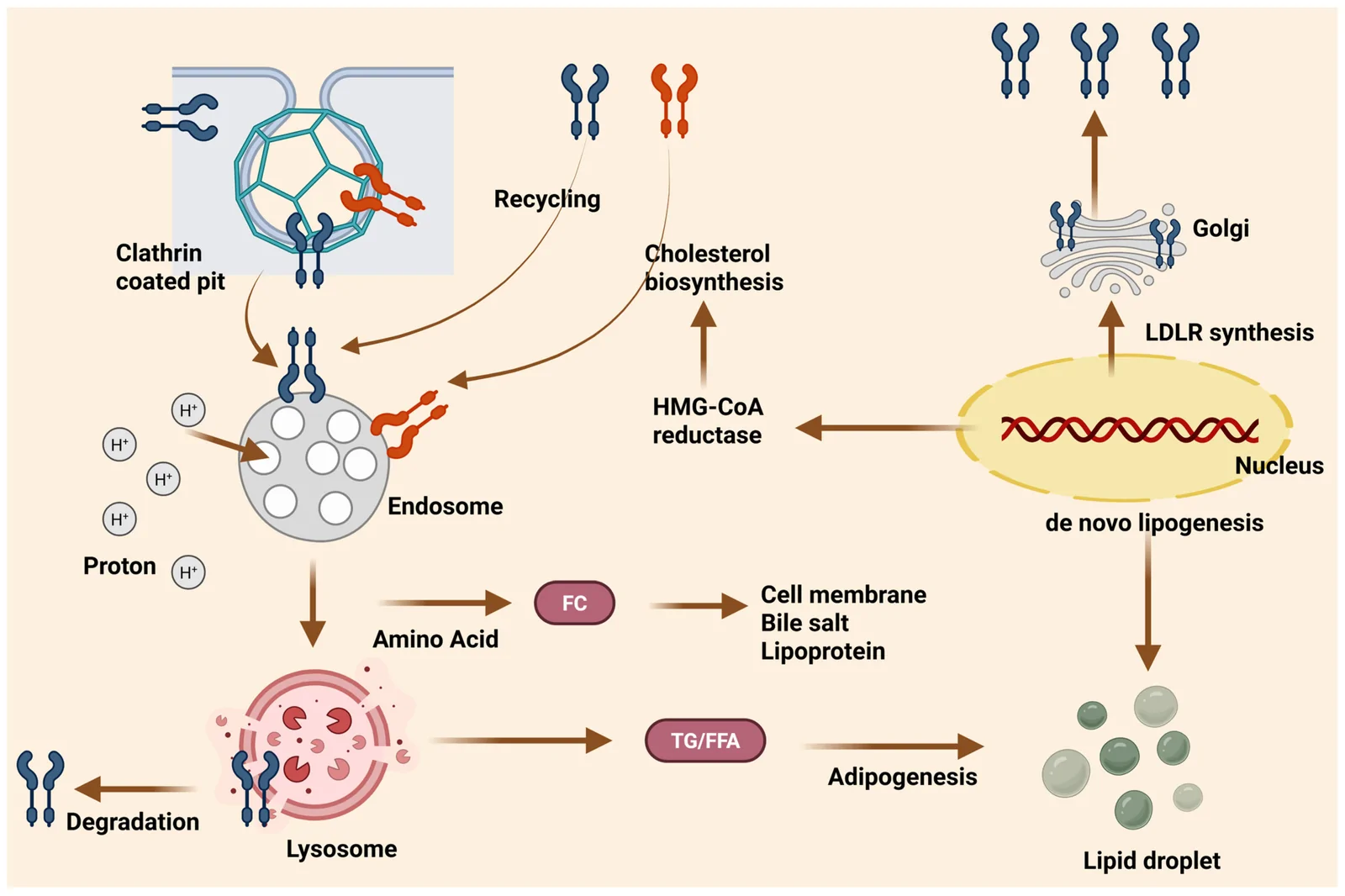
Cellular uptake and internalization pathways in PC. Created in BioRender. Hani, U. (2026) https://BioRender.com/aqd0lri (accessed on 14 June 2026).

AuNPs are generally able to pass through the endosomal and lysosomal compartments after entering the cells. For many stimuli-responsive systems, however, it is more favorable for such materials to escape the endosome due to its ability of providing delivery to cytoplasm and facilitating drug or genetic material availability. Notably, since endosomal escape is critical for gene delivery and other therapeutic applications, cellular uptake must be accompanied with effective endosomal escape to optimize therapeutic potency (Figure 4). Hence, knowledge of cellular uptake and internalization pathways is crucial for improved optimized design of nanoparticles, further development of intracellular drug delivery technologies in the clinical setting, and increased therapeutic effectiveness in prostate cancer nanomedicine [82].

### 5.2. Induction of Apoptosis and Cell Cycle Arrest

AuNPs cause apoptosis and cell cycle disruption in prostate cancer therapy, which results in induction of anti-tumor growth and proliferation inhibition. The direct impact on the cell may concern nanoparticle–cell interaction, whereas indirect effects are linked with enhanced uptake of cytotoxic drugs/photothermal therapy or radiosensitizers. A major mechanism is via activation of the intrinsic (mitochondrial) apoptotic pathway [83]. The AuNPs can also lower the level of ROS generation, which causes depolarization of mitochondrial membrane, release of cytochrome c and subsequent activation of caspase-9/caspase-3. This cascade culminates in DNA fragmentation and then apoptotic cell death. The prostate cancer cells which have been treated with AuNPs based systems showed the upregulation of pro-apoptotic proteins (Bax) and downregulation of anti-apoptotic proteins (Bcl-2). Furthermore, interfacing of functionalized AuNPs with cell surface death receptors can trigger the extrinsic apoptotic pathway. This pathway is mediated through caspase-8, which amplifies the downstream apoptotic signaling.

Besides facilitating apoptosis, the capability of gold nanoparticles to inhibit cancer cells is brought about through cell cycle arrest—preventing them from progressing at critical checkpoints required for replication. The earlier studies also showed that AuNPs induce arrest at the G0/G1, S or G2/M phases by using recent data dependent on parameters which include particle size, surface modification and treatment modality. This arrest is often correlated with the regulation of primary regulatory proteins such as cyclins, cyclin-dependent kinases (CDKs) and p53-proteins, along with their suppressors. AuNPs cause a significant inhibition in the proliferation of tumor cells owing to interference with DNA replication and mitosis [84].

### 5.3. Production of Reactive Oxygen Species (ROS)

The generation of ROS induced by gold nanoparticles (AuNPs) is an important mechanism by which AuNPs exhibit anticancer effects for prostate cancer treatment. ROS are byproducts of normal oxygen metabolism that include highly reactive molecules like superoxide anions (O_2_^−^), hydrogen peroxide (H_2_O_2_) and hydroxyl radicals (•OH). Gold acts as a direct redox and may activate survival apoptosis by way of sonophoresis; alternatively, it raises the production of ROS inside cells or enhances coadjutant therapeutic modalities. Gold is radiolucent due to its high atomic number, resulting in numerous ionization events which are responsible for the generation of secondary electrons, making a significant contribution to ROS [85]. Similarly, on photothermal initiation by near-infrared (NIR) irradiation, hyperthermia mediated locally could intervene in mitochondrial function and subsequently induce more ROS.

Excessive generation of ROS leads to oxidative stress, which is detrimental to key cellular elements, including lipids, proteins and nucleic acids. However, a high concentration of ROS in prostate cancer cells might disturb the redox homeostasis, causing mitochondrial membrane depolarization and DNA strand breakage, as well as activating stress-responsive signaling pathways involving p53 and MAPK or JNK. Ultimately, these events lead to apoptosis or other forms of programmed cell death.

In particular, cancer cells often exist under elevated basal oxidative stress and therefore are more susceptible to further ROS-induced injury. AuNP-mediated ROS amplification can then enhance the cytotoxicity preferentially in tumor cells, which have a regulated mitochondrial membrane potential (Δψm), more so than normal tissues. Moreover, systemic induction of excessive oxidative stress leads to off-target toxicity despite modulation of ROS being closely governed at the level of cellular organelle redox. Thus, enhancing the therapeutic efficacy of target-specific and ROS-generating agents without losing minimization of side effects is possible only when gold nanoparticle systems are designed as optimal synergistic agents towards them [86]. Thus, these findings suggest that the improvements of anticancer activity by gold nanoparticles are being achieved through a key molecular mechanism, and ROS generation is acting as an auxiliary method in prostate cancer treatment with respect to diverse methods like radiotherapy, chemotherapy or photothermal-based methods.

### 5.4. Anti-Angiogenic and Anti-Metastatic Effects

Their extraordinary anti-angiogenic and anti-metastatic potentials have led to improved prospects for the use of AuNPs as therapeutic vectors for prostate cancer. Angiogenesis plays an important role for cancer cells’ morphological proliferation with oxygen and nutrients, which leads the tumor progression and metastasis. AuNPs are capable of blocking tumor growth and metastasis.

The anti-angiogenic mechanism of AuNPs is associated with the inhibition of VEGF signaling pathways. Gold nanoparticles similar to heparin could attach to the heparin-binding domain of VEGF, blocking the interaction between VEGF and its cognate receptors (VEGFR) on endothelial cells. Neovascularization in the tumor microenvironment was also inhibited by the inhibiting of proliferation, migration and tube formation of endothelial cells. Furthermore, AuNPs can downregulate pro-angiogenic genes and therapeutic pathways such as PI3K/Akt and MAPK, ultimately leading to defective vascular maturation.

In respect to anti-metastatic activity, AuNPs have been shown to interfere with relevant mechanisms of tumor cell invasion and migration. So far, it has been found by researchers that they inhibit matrix metalloproteinases (MMPs), enzymes that cleave the components of the extracellular matrix and promote the invasion of tumor cells. AuNPs inhibit the expression and activity of MMPs, leading to diminished tissue penetration of prostate cancer cells as well as migration into blood circulation. In addition, AuNP therapy has been cited to reduce epithelial–mesenchymal transition (EMT), a critical step in the metastatic cascade; this is evidenced by resumed expression of epithelial markers and decreased mesenchymal marker expression [87].

As to drug delivery aspects, AuNPs can also serve as specific carriers in order to deliver antiangiogenic agents or chemotherapy drugs, neutralizing tumor spread. Together with photothermal therapy or radiotherapy, they can induce further destruction of tumor vasculature and also possibly lower metastatic potential.

### 5.5. Radio Sensitization Mechanism

This is thought to be mainly due to the-atomic number of Au and its high ability to absorb X-rays, which has led to a higher local deposition of radiation dose in cancer tissue. Ionizing radiation (X-rays or γ-ray) exposes AuNPs to secondary electrons such as photoelectrons, Auger electrons, and Compton electrons generated by photoelectric and Compton interaction. These low-energy electrons deposit energy over micrometer ranges and can cause localized ionization, leading to enhanced DNA damage in neighboring tumor cells. This results in heightened generation of non-negotiable lethal damage, i.e., single-strand and double-strand DNA breaks [88].

Another mechanism is amplification of reactive oxygen species (ROS). Radiation also interacts with water molecules, generating hydroxyl radicals (•OH) and other ROS. AuNPs exhibited an enhanced radiolytic effect and contributed to higher oxidative stress. Increased ROS levels lead to cytotoxicity of prostate cancer cells via damaging their DNA and proteins, and compromising cell membranes. AuNPs further regulate the biological pathways involved in responses to radiotherapy. They may affect DNA repair pathways, resulting in a lag time for clearing damaged DNA. Moreover, AuNP-induced oxidative stress can activate apoptotic signaling pathways and lead to the impairment of cell cycle progression, particularly for cells in G2/M phase, since it is the most sensitive to radiation [89].

Only tumor tissue undergoes selective aggregation of gold nanoparticles, increasing the radiation effect while reducing damage to surrounding healthy tissue; allowing for the combination of both mechanisms. This novel tumor-targeted dose enhancement not only improves therapeutic efficacy but may also reduce the radiation dose required for treatment and therefore reduce systemic side effects. In summary, gold nanoparticles’ radiosensitization modes may be explained through physical dose enhancement and generation of secondary electrons, amplification of ROS and alteration of cellular repair pathways. They are also a very powerful adjuvant to enhance the therapeutic efficacy of prostate cancer radiotherapy [90].

#### 5.5.1. Mechanisms of Radiation Dose Enhancement

AuNPs cause an enhanced radiosensitization effect mainly due to their capacity for local radiation energy deposition in tumor tissues. Due to the high atomic number and electron density of gold, AuNPs have a much higher X-ray absorption coefficient than biological tissues. Gold nanoparticles preferentially absorb incoming photons due to the photoelectric effect and this becomes significant in the presence of low and medium energy X-rays. This interaction generates photoelectrons and Auger electrons of high energy that deposit their energy very close to the vicinity of these nanoparticles.

The localized production of these secondary electrons leads to micro-scale amplification of the radiation dose near the ionizing source, resulting in extensive ionization of neighboring biomolecules. Consequently, the tumor cells loaded with AuNPs will receive much higher intracellular radiation dose than non-targeted normal tissues surrounding the tumor, enhancing the therapeutic ratio of radiotherapy.

#### 5.5.2. Generation of Reactive Oxygen Species

Increase of reactive oxygen species (ROS) generation induced by AuNP-mediated radiation enhancement is one of the well-known biological effects. Ionization of the cell during irradiation leads to secondary electrons which in turn will interact with intracellular water molecules, giving rise to some oxidative stress such as hydroxyl radicals (•OH), superoxide radicals (O_2_•^−^), hydrogen peroxide (H_2_O_2_) and other highly reactive intermediates.

These ROS ultimately lead to oxidative damage to the lipids, proteins and nucleic acids, disrupting essential cellular functions. Elevated oxidative stress further leads to mitochondrial dysfunction, loss of anti-apoptotic protein bcl-2 level and mitochondrial transmembrane potential with cytochrome c release/direct activation of caspase-dependent apoptotic pathways [7]. Thus, AuNP-mediated ROS amplification significantly enhances the radiation-induced cytotoxicity in tumors.

#### 5.5.3. DNA Damage and Blocking DNA Repair

DNA is the major intracellular target for radiotherapy. Increased yields of secondary electrons and ROS from AuNP irradiation led to a dramatic rise in human cellular DNA double-strand break (DSB) formation, the most lethal type of radiation-induced DNA damage.

Several reports have shown that AuNPs cause not only increased DNA damage, but also disruption of the cellular DNA repair machinery. Exposure to AuNP may inhibit the activation of homologous recombination (HR) and non-homologous end joining (NHEJ) pathways induced by radiation so that repair of DNA double-strand breaks is not efficient. The persistent DNA damage in turn activates p53-dependent apoptosis, mitotic catastrophe and irreversible cell-cycle arrest, inducing a greater tumor control.

#### 5.5.4. Cell Cycle Modulation

Exposure to ionizing radiation is particularly lethal during the G2/M phase of the cell cycle, making cellular radiosensitivity phase-dependent. Many studies have shown that AuNP induces cell-cycle arrest at the G_2_/M checkpoint, and thus synchronizes tumor cells at their most radiosensitive phase prior to irradiation. This could also be part of the mechanism for the observed synergistic effects when AuNP is combined with radiotherapy, as it serves to determine augment radiation induced cytotoxicity.

#### 5.5.5. Tumor Microenvironment and Radiosensitization

In addition to the direct repair of radiation damage, AuNPs can also affect the tumor microenvironment. Radiation in combination with AuNPs has been demonstrated to reduce the tumor-induced angiogenesis, increase vascular permeability, and facilitate oxygen diffusion into hypoxic regions of tumors. The result in improved oxygenation enhances the radiation induced ROS formation, due to molecular oxygen preventing DNA repair by stabilizing the free radical sites of DNA. Additionally, AuNP-mediated modulation in inflammatory signaling and cytokine production may also play a role in the enhancement of immune-mediated tumor clearance after radiotherapy.

#### 5.5.6. Targeted Radiosensitization

The radiosensitizing efficacy of AuNPs can be enhanced even further via surface functionalization to achieve selective tumor accumulation. Functionalization of gold with prostate-specific membrane antigen (PSMA)-targeting ligands, antibodies, peptides, or aptamers induces receptor-mediated internalization by prostate cancer cells and enhances intracellular concentration of gold prior to irradiation. PEGylation prolongs systemic circulation and prevents non-specific uptake by the reticuloendothelial system, thereby providing tumor-selective radiosensitization in a single step.

Recently developed multifunctional nanoplatforms combine radiosensitization with chemotherapeutic agents, photothermal therapy, molecular imaging and gene delivery for mutual diagnostic, and therapeutic purposes in a single theranostic system.

### 5.6. Photothermal and Photodynamic Therapy

AuNPs are gaining a lot of interest in prostate cancer treatment through the means of photothermal mediated therapy (PTT), as well as aiding in development of photodynamic therapy (PDT); both are minimally invasive and specially controllable.

#### 5.6.1. Photothermal Therapy (PTT)

Nanoparticle size, shape and structure dictates the efficiency of photothermal transformation. These GNPs are shown by hybrids of gold nanorods, nanoshells and/or nanostars with tunable SPR maxima in the NIR range to enable deep tissue penetration and selective ablation of the tumor. Critically, PTT enables spatially-concentrated thermal damage: only those areas that are under radiation and contain nanoparticles become damaged, while nearby healthy tissue remains intact [91].

#### 5.6.2. Photodynamic Therapy (PDT)

Photodynamic therapy (PDT) is based on photosensitizers, which produce reactive oxygen species (ROS), particularly singlet oxygen (^1^O_2_), upon activation by light at a specific wavelength. Since gold nanoparticles are not classical photosensitizers, this can also serve to enhance PDT via different ways: AuNPs are also promising candidates that have been developed as carriers in vitro since they efficiently improve the tumor accumulation and stability of the photosensitizing agent. Additionally, the plasmonic nature of these metals can amplify local electromagnetic fields that stimulate ROS production. Upon mediating PDT, application of AuNP results in high oxidative stress-induced cellular injury, mitochondrial dysfunction, DNA breakage, and apoptosis in prostate cancer. Synergistic therapeutic efficacy between PDT and PTT often is produced through hyperthermia to promote ROS-mediated cytotoxicity [92].

#### 5.6.3. Combined Phototherapy (PTT + PDT)

The realization of simultaneous photothermal and photodynamic activation endowed a multifunctional gold nanoplatform for eradication of tumors. The heterogeneous mechanism of invasion used localized heating to ROS oxidative damage responses; these strongly promoted the complete destruction of cancer cells, thereby enhancing recurrence inhibition. Gold nanoparticle-mediated photothermal and photodynamic therapies are highly effective, low invasive approaches with enhanced tumor specificity, adjustable activation and excellent potential for integration into precision prostate cancer therapy [93].

#### 5.6.4. Mechanism of Heat Generation

The main mechanism for the photothermal effect of AuNPs is localized surface plasmon resonance (LSPR). The plasmon resonance of AuNPs is activated when light with a wavelength corresponding to the particle shape oscillates collectively as conduction-band electrons on the surface of a nanoparticle. This efficiently enables the absorption of optical energy in a wide range of wavelengths, from visible light all the way to the near-infrared (NIR) biological window (700–900 nm), where the tissue absorption through water and hemoglobin is low, enabling deep penetration of even longer wavelengths into tissues. Immediately after photon absorption, excited electrons rapidly relax via electron–electron scattering on the order of femtoseconds and then undergo electron–phonon coupling within hundreds of femtoseconds to transfer the absorbed energy from electrons to the nanoparticle’s crystal lattice. The energy is then lost to the surrounding tissue as heat via phonon–phonon relaxation within picoseconds to nanoseconds. This can result in localized temperatures 45–60 °C as rapid heating of tissues leads to very spatially confined thermal damage with limited effects on surrounding normal cells.

#### 5.6.5. Mechanisms of Tumor Ablation

Contrastingly, localized hyperthermia caused by AuNPs is a complex biological phenomena leading to destruction of tumor cells through coordination of multiple complemental mechanisms and not just due to thermal injury. Increased intracellular temperature leads to irreversible denaturation of protein, cytoskeleton disturbances, mitochondrial dysfunctions, membrane destabilization and DNA damage that can inhibit metabolic enzymes. Such cellular changes ultimately lead to the activation of intrinsic apoptotic pathways via mitochondrial cytochrome c liberation, caspase cascade and DNA fragmentation. Rapid coagulative necrosis occurs with oven-like temperatures >50 °C; this can refer to membrane rupture, cytoplasmic protein coagulation and irreversible enzyme inactivation. In parallel, thermal injury causes damage to tumor-specific vasculature leading to vascular collapse, which impairs oxygen and nutrient delivery, thus enhancing tumor cell death.

#### 5.6.6. Combination Treatment Strategies

AuNPs used as both drug carriers and photothermal agents allow simultaneous chemotherapy and thermal ablation. Localized heating helps to accelerate the drug release from temperature-sensitive nanocarriers, enhances the permeability of membranes, and strengthens intracellular uptake of drugs. Therefore, co-administration of chemotherapeutic agents, including docetaxel, doxorubicin, paclitaxel or cisplatin, often achieves synergetic antitumor effects with the opportunity to reduce doses and toxicity in normal tissues. One such approach is photothermal therapy, where local immunologically “cold” tumors can serve as a source of new microscopically active lesions to augment the immune response from immunotherapy. Thermal ablation stimulates the release of tumor-associated antigens and death-associated molecular patterns which promote dendritic cell activation, as well as T-cell priming supporting improved immunity to immune checkpoint inhibitors targeting PD-1, PD-L1 or CTLA-4. Inhibitors of immune checkpoint and photothermal therapy delivered by multifunctional AuNPs have shown preclinical promise as antitumor therapeutic agents. AuNPs can be used as dual-functional agents that deliver nucleic acids (siRNA, miRNA, or CRISPR-associated gene-editing systems) and heat simultaneously. Hyperthermia improves the intracellular delivery, endosomal escape and gene silencing by nucleic acid therapeutics. These combination approaches engender avenues for concurrent blockade of oncogenic signaling pathways and adjuvant thermal ablation of neoplastic cells.

## 6. Gold Nanoparticle Mediation: Emerging Roles in Prostate Cancer Immunotherapy

**Figure 5:**
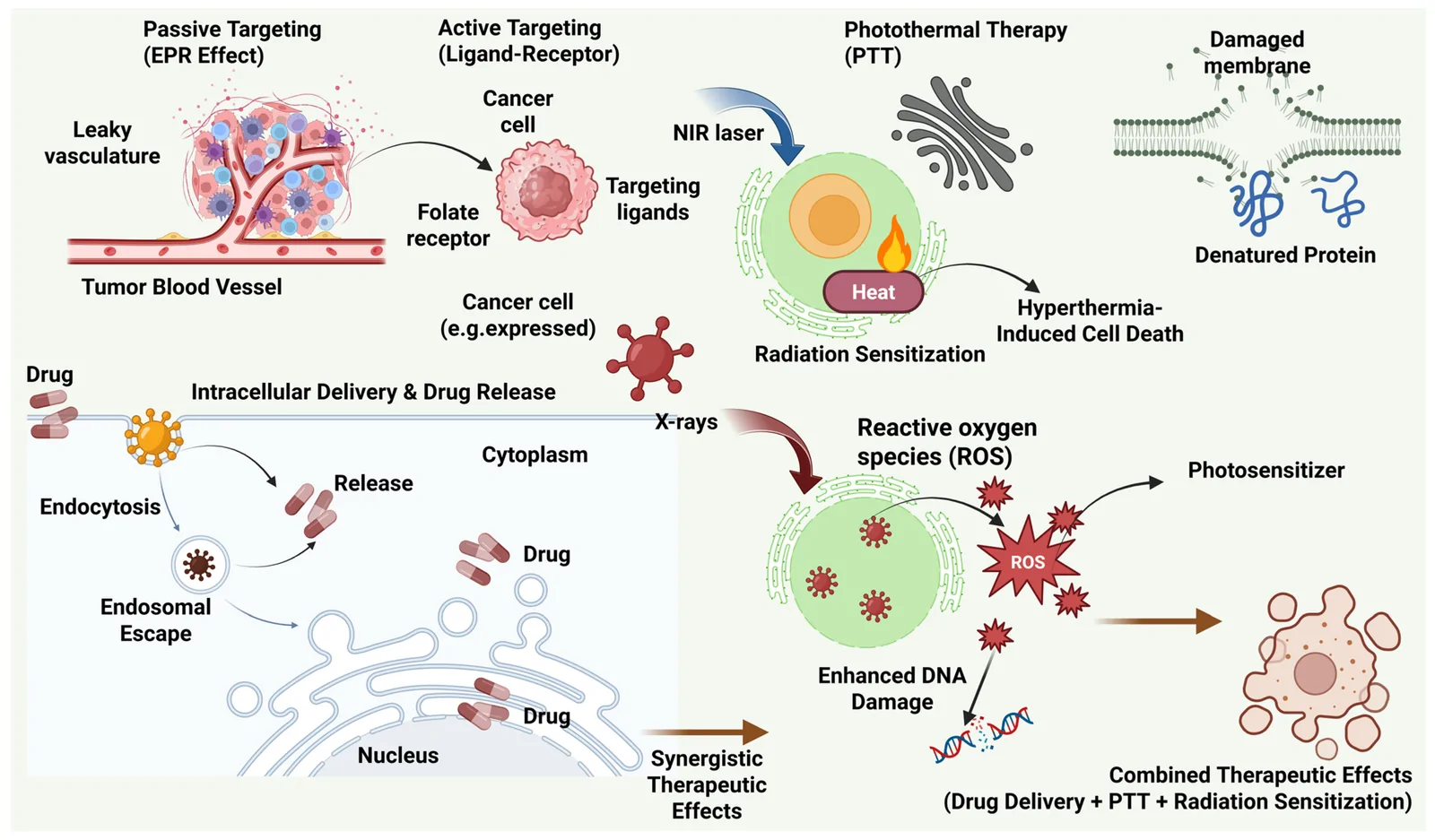
Gold knanoparticle-mediated drug delivery and therapeutic mechanisms. Created in BioRender. Hani, U. (2026) https://BioRender.com/ejxgc0f (accessed on 14 June 2026).

AuNPs exert immune responses via distinct mechanisms, such as the modulation of innate immune cells, regulation of cytokine production, enhancement of antigen presentation and adaptive immunity, promoting activation. The high surface area can also support the simultaneous conjugation of tumor antigens, adjuvants, immune checkpoint inhibitors, cytokines and targeting ligands to develop multifunctional immunotherapeutic nanoplatforms. Moreover, the physicochemical properties of AuNPs (e.g., particle size, morphology and surface chemistry) play a critical role in their interaction with immune cells as well as immunological outcomes. Gold nanoparticle mediated drug delivery and therapeutic mechanisms are depicted in in Figure 5.

### 6.1. Macrophage Polarization

Tumor-associated macrophages (TAMs) represent one of the most abundant immune cell types in the prostate tumor microenvironment. The majority of TAMs display an M2-like phenotype that facilitates tumor growth via angiogenesis, extracellular matrix remodeling, and immune suppression by releasing anti-inflammatory cytokines, including interleukin-10 (IL-10), and transforming growth factor-beta (TGF-β). Multiple studies demonstrate that engineered AuNPs can skew macrophage polarization from the tumor-supportive M2 phenotype to the pro-inflammatory M1 phenotype. M1 macrophages secrete tumor necrosis factor-α (OH-α), interleukin-12 (IL-12), reactive oxygen species (ROS), and nitric oxide, leading to increased tumor cell destruction and reflexive T cell-induced immune stimulation. Coating AuNPs with immunostimulatory molecules or pathogen-associated molecular patterns (PAMPs) significantly augments the activation of macrophages and promotes antitumor immunity.

### 6.2. Activation of Dendritic Cells

Dendritic cells (DCs) are considered professional antigen-presenting cells that initiate adaptive immune responses by activating naïve T lymphocytes. We also demonstrate that auNPs efficiently deliver tumor-associated antigens and immunostimulatory adjuvants to dendritic cells, enhancing antigen uptake, maturation and cross-presentation via major histocompatibility complex (MHC) molecules. These activated dendritic cells then activate CD8^+^ cytotoxic T lymphocytes specific to the API peptide, which are capable of recognizing and killing prostate cancer cells. AuNPs are of great interest as vaccine delivery vehicles for therapeutic cancer vaccination, because they protect peptide antigens from enzymatic degradation and promote efficient intracellular delivery.

### 6.3. Regulation of T-Lymphocyte Responses

The activation and proliferation of CD8^+^ T cytotoxic cells is a required component in effective immunity against tumors. In this regard, AuNPs can also indirectly facilitate T-cell responses by improving antigen presentation and decreasing immunosuppressive signaling in the tumor microenvironment. In addition, cytokine delivery using nanoparticles (NPs), including interleukin-2 (IL-2), interleukin-12 (IL-12) or interferon-γ(IFN-γ), has been established to stimulate tumor-specific T cell proliferation and activation with lower systemic toxicity by cytokines. Other recent studies have investigated AuNP-mediated delivery of small interfering (si-) RNA targeting immunosuppressive pathways in tumor cells, thus restoring immune recognition to improve T-cell mediated cytotoxicity.

### 6.4. Synergistic Augmentation of Immune Checkpoint Benefit

Immune checkpoint molecules such as programmed cell death protein-1 (PD-1), programmed death ligand-1 (PD-L1) and cytotoxic T-lymphocyte-associated protein-4(CTLA-4) downregulate T-cell activation, thereby suppressing antitumor immune responses. Despite the exceptional clinical success of immune checkpoint inhibitors for cancer treatment, their use in prostate cancer is limited by poor infiltration of active immune cells and an immunosuppressive tumor microenvironment. AuNPs represent an appealing platform for actively targeting the delivery of immune checkpoint inhibitors to tumoral tissues. Conjugation of anti-PD-L1 or anti-CTLA-4 antibodies onto AuNPs increases the amount of drug at the site and decreases systemic immune-related adverse events. Additionally, nanoparticles can achieve a co-delivery of immune checkpoint inhibitors with chemotherapeutic agents or photothermal therapy and have exerted synergistically antitumor immunity in preclinical studies.

## 7. Gold Nanoparticles in Prostate Cancer Diagnosis

Based on their unique optical properties and surface functionalization approaches, AuNPs have been developed into attractive tools for prostate cancer diagnostics. Due to their enhanced SPR, high surface-to-volume ratio, and some immune properties, they are promising candidates for biosensing and molecular imaging applications and early tumor detection. This allows the functionalization of AuNPs with antibodies, aptamers or peptides that bind specific aspects, assist in changing their optical or electrical characteristics. Colorimetric assays have a rapid visual color change from red into blue due to AuNP aggregation, which enables sensitive detection of PSA. This principle can also be utilized by plasmon-enhanced Raman and spectroscopic (SERS) substrates such as gold-based nano-sensors for the highly sensitive identification of trace biomolecules [94].

Due to their high atomic number, AuNPs are promising contrast agents for CT imaging as they can attenuate X-rays to a greater extent than conventional iodinated contrast agents. This adds to better visualization and demarcation of the tumor. They can also be useful for profiling the absorbance to light and generation of an ultrasonic wave when imaging a tumor for photoacoustic imaging (PAI), which is good for resolution [64,66]. This conjugation has led to multimodal imaging strategies with fluorescent dyes or radioactive tracers that enable precise localization at the site of tumors. Of these solid tumors, prostate cancer has focused on AuNPs functionalized with PSMA ligands or receptor ligands to selectively bind it in vivo [8]. This enables early detection on the molecular scale, which lends diagnostic precision. Because the nanoparticles have a nanoscale size, these particles could reach to tumor tissues and upregulate signals in a disease-associated areas [95].

### 7.1. AuNPs-Based Biosensors

The AuNPs-based biosensors characterized in several of the studies demonstrated high sensitivity and selectivity, indicating that these nanomaterials show promise for uses as diagnostic platforms for the early diagnosis and monitoring of prostate cancer. Depending on their photoelectric, electrical conductivity and surface area properties, MNPs are an ideal candidate for use in detection methods [96]. One of the most popular applications is the PSA (Prostate-Specific Antigen) detection performed in clinical diagnostics as a prostate cancer screening biomarker. In colorimetric biosensing, AuNPs aggregation causes a change in the color of their photon absorbance from red to blue. These reagents are functionalized to have either antibodies or aptamers that specifically target PSA bind to them, inducing the binding event at the surface level and resultant aggregation or end dispersal of nanoparticles as a color change and useable under spectrophotometry. Such assays are simple, rapid, low cost and POCT. The good electrical conductivity of gold provides a significant amplification of the signal for electrochemical–AuNP based biosensors. AuNPs can be deposited onto the surfaces of electrodes and are involved in enhancing electron transfer kinetics as well as providing a much larger surface area for immobilization of biomolecules. This leads to an enhanced PSA, PSMA or other tumor-associated marker sensitivity with decreased detection values.

SERS-type biosensors often utilize the plasmonic properties of AuNPs to enhance Raman signals from bio-molecules adsorbed onto their surfaces. Its application lies in ultra-sensitive detection of low concentration of cancer biomarkers, which contributes to early-stage diagnosis. Antibodies, peptides, or aptamers can be conjugated on to their surface for high specific molecular recognition. There are many different techniques and technologies used to develop sensitive diagnostic biosensors based on AuNP as a result of specific detection parts. These benefits have considerable implications for the use of liquid biopsy technologies in early detection, prognostication and disease monitoring throughout prostate cancer treatment.

### 7.2. Imaging Applications

Gold nanoparticles (AuNPs) showing unique optical and physicochemical characteristics, are promising as multimodal imaging agents for prostate cancer diagnosis. Nanoparticles can provide high-atomic-number tunable surface chemistry, and their nanoparticles have a high surface area for enhanced sensitivity by the interaction with SPR effects to promote use as contrast-enhancing imaging agents in increasing tumor visualization and signal across multiple imaging modalities [97].

#### 7.2.1. Computed Tomography (CT)

Owing to its far higher X-ray-absorption coefficient relative to the conventional iodine-based contrast agents, AuNPs have been proposed as potential contrast agents in X-ray dynamic computed tomography imaging. This provides greater contrast and clearer separation of the tumors. In addition, the intratumor accumulation and circulation times of AuNPs are also known to be increased by steady passive EPR effect or active targeted strategies. Surface-functionalized AuNPs that target prostate-specific membrane antigen (PSMA can also be exploited to enhance specific imaging of the prostate tumors for better localization and improved diagnostic accuracy [98].

#### 7.2.2. Magnetic Resonance Imaging (MRI)

Because of its non-magnetic property, AuNPs can be assembled with iron oxide materials to create a hybrid nanocomposite. These multi-modal platforms can deliver two different imaging modalities: CT contrast, from the gold component, and MRI contrast from the magnetic components. These systems aid in soft tissue visualization, spatial resolution and tumor characterization. Successful matching of surface-modified tumor-homing ligands enhances additional tumor-localization, thereby augmenting the MRI signal at the disease sites [99].

#### 7.2.3. Optical Imaging

The SPR and light-scattering properties of gold nanoparticles are promising, so they play a useful role in optical imaging. Over the past decade, these materials have been studied and explored in photoacoustic imaging (PAI), a novel imaging method which employs light absorption to generate ultrasonic signals for high-resolution detection of deep tissue. The emission intensity of certain fluorescent dyes can be enhanced by AuNPs, which enables us to obtain better results in fluorescence imaging. Accurate and ultrasensitive molecular detection at low biomarker concentrations can be achieved with SERS-based imaging [100].

#### 7.2.4. Multimodal Imaging Potential

One of the great promises of AuNPs is the availability of multi-modality imaging on a single platform (i.e., a CT/photoacoustic or CT/MRI system). This multimodal nature allows for comprehensive characterization of tumors at the anatomical, functional and molecular levels. So, most importantly, imaging systems exploiting footfall size ranges of gold nanoparticles will offer contrast enhancement upon signature features found specifically in ECPs thereby rendering an improved specificity towards active tumor cells of TME but also potentially allowing (specific to applications) improvement during early detection of formalin from recurrent disease and thus they can be strategically highlighted as optimal platforms for incorporating precision diagnostic oncology.

### 7.3. PSA Detection Enhancement

In PSA detection, gold nanoparticles (AuNPs) are commonly used to improve specific and reliable detection techniques, Because of this, the sensitivity of such techniques has increased in recent years. Standard approaches for PSA detection like enzyme-linked immunosorbent assay (ELISA) suffer limitations in low antigen concentration sensitivity and self-activation in complex biological backgrounds. Thus, AuNPs have been previously used on diagnostic platforms to overcome these barriers since they can amplify both molecular coverage and signals in a detection signal. This is most notably demonstrated with colorimetric tests utilizing SPR AuNPs wherein conjugation of the nanoparticle results in a colorful red to blue transition. By functionalizing with anti-PSA antibodies or PSA-specific aptamers, binding of PSA triggered either formation of aggregates (aggregation) or prevented salt-induced aggregation (disaggregation); both processes produced quantifiable optical readouts. This allows rapid, economic and highly sensitive input at low PSA levels [101].

The immobilization of AuNPs on electrode surfaces expands the surface area and improves kinetics in electron transfer reactions as applied to electrochemical-type biosensors. Signal transduction with a high sensitivity and better analytics properties is supported via the e−conduction evoked in the sensing units. They could also sense PSA levels down to the picogram per milliliter (pg/mL) range, assisting in the identification and diagnosing early stages. Moreover, researchers are more specifically interested in improvements mentioned in the SERS-relevant literature around platforms that observe such an enhancement of PSA detection resulting from their use of the plasmonic properties of AuNPs to amplify the Raman signals arising from biomolecules bound to its surface. This strategy allows ultrahigh sensitivity and specificity of molecular fingerprinting of PSA down to trace concentrations [102].

## 8. Gold Nanoparticles for Targeted Drug Delivery

The significance of this research is primarily due to the rise in PC rates worldwide and limited treatment, which have pushed scientists to find novel therapies not only with new medications, but also new targeted delivery systems like AuNPs that have already shown potential, but without specificity. The tunable size, high surface area, and good biocompatibility along with easy surface functionalization have made AuNPs an effective drug delivery vehicle for therapeutic compounds, as per the preclinical study on the PC model. These properties enable the specific delivery of chemotherapeutic agents as well as genes or biomolecules directly to a tumor cell site with minimal systemic toxicity [103]. One of the most important attributes of AuNPs, having small size compared to other kinds of nanoparticles, is that they can be more successfully localized in tumor tissues (solid tumors), due to the enhanced permeability and retention (EPR) effect. Moreover, AuNPs could be engineered for active targeting through covalent conjugation with a great variety of ligands including antibodies, peptides or aptamers against prostate cancer-specific biomarkers conserved in prostate malignancy as PSMA. Utilization of receptor–ligand interaction through endocytosis can exert selective uptake and intracellular delivery of a drug inside malignant cells.

In general, there are various drug loading techniques utilizing AuNPs based on the desired technique (e.g., physical adsorption, covalent attachment (thiol–gold chemistry), and encapsulation in polymer-coated nanostructures). Stimuli-smart linkers drive controlled drug release to the TME, mostly involving pH-sensitive or redox-sensitive bonds due to the low pH and glutathione-rich environment of a tumor. This enables localized release and reduces off-target effects. The AuNPs serve as both the carriers of small-molecule chemotherapeutics and nucleic acids (e.g., siRNA, miRNA and plasmid DNA) for silencing or restoration therapies. Multifunctional AuNP systems are expected to matter for theranostics, as they can potentially enable localized drug delivery and imaging or photothermal therapy at the same time. They are ideal nanocarriers in that they molecularly target drug delivery for prostate cancer therapy, achieving increased drug stability, better tumor targeting and prolonged release time, which effectively lowers systemic toxicity [104].

### 8.1. Loading of Chemotherapeutic Agents

One of the most favorable applications concerning the use of this approach through nanotechnology to enhance clinical outcomes in prostate cancer patients includes the use of chemotherapeutic drugs on AuNPs drug loading chemotherapeutic agents including Docetaxel. Docetaxel is a first-in-class taxane chemotherapeutic agent approved for the treatment of metastatic and castration-resistant prostate cancer. Docetaxel stops the breakdown of mitotic spindle and causes cell cycle G2/M phase arrest, which leads to apoptosis; its anti-neoplastic effect is achieved by preventing the disassembly of microtubules. Docetaxel has been a successful drug in clinical trials and afterwards; at the same time its use has been considerably hampered due to aqueous insolubility requiring toxic solubilizing agents like polysorbate 80, rapid clearance from systemic circulation, multiorgan distribution, evolution of multidrug resistance and side effects including neutropin peripheral neuropathies (dose limiting). Furthermore, facilitating drug delivery in suitable AuNP-based systems such as ideal solubilizing reduces most of these limitations by enhanced pharmacokinetic profiles and tumor-targeted accumulation. A physical adsorption receptor utilizing a hydrophobic or electrostatic interface enables adsorption loading, but desorbs rapidly [105].

Polymers containing –COOH or –NH_2_ groups attached to surface-functionalized AuNPs are often simplified by delivery through the use of a variety of coupling agents, including amide bond formations with EDC/NHS, which is otherwise achieved via covalent conjugation such as thiol–gold chemistry. Furthermore, polymer-assisted strategies like PEGylating or chitosan coating allow for the encapsulation of docetaxel in a biocompatible layer surrounding the gold core with improved colloidal stability, higher systemic circulation half-life and immune recognition while promoting sustained drug-release. More advanced systems use stimuli-responsive linkers which are cleavable at acidic pH or high levels of glutathione in the tumoral microenvironment, which guarantees controlled intracellular drug release [106]. Also, by conjugating targeting ligands (antibodies, peptides or aptamers) against prostate-specific membrane antigen (PSMA), the process of receptor-mediated endocytosis is enhanced, leading to augmentation of intracellular drug concentration in prostate cancer cells. Passive tumor targeting is further aided by the enhanced permeability and retention (EPR) effect. Using these multimodal AuNPs-based docetaxel therapeutic platforms, we show markedly enhanced cytotoxic effects with evasion of known mechanisms of resistance while significantly reducing systemic toxicity and improving the overall cost–benefit ratio; thereby reiterating their potential to influence precision nanomedicine paradigms in prostate cancer therapy.

### 8.2. Gene Therapy and Small Interfering RNA Delivery

Out of all the carriers AuNPs have shown to be very effective and versatile in gene therapy, particularly prostate cancer treatment using small interfering RNA (siRNA). Evidence for the novel carrier design extends over 5 years. This may involve gene therapy to correct or silence abnormal expression of a gene, or the delivery, silencing and/or editing of specific genes that are known to be under- or over-expressed in the tumor. Naked nucleic acids (siRNA or plasmid DNA) are prone to enzymatic degradation, show poor cellular uptake rate, are rapidly cleared and display low stability intracellularly. Error-prone delivery of genetic material can be circumvented through enhanced AuNP-based complexes that act as protective components, followed by translocation into the cells. Functionalization with thiol-modified oligonucleotides on AuNPs occurs through stable gold–sulfur (Au–S) interactions, leading to highly stable nanoconjugates. In contrast, positive surface coatings (such as polyethyleneimine or chitosan) can electrostatically attach to negatively charged nucleic acid and promote active siRNA loading. These changes improve serum stability, but they also prevent nuclease-mediated degradation in the circulation.

AUNP in prostate cancer has also been investigated for the delivery of siRNA-related therapies to oncogenes including forms of androgen receptor signaling or anti-apoptotic proteins (an example being Bcl-2) or with pathway stepping such as the PI3K/Akt pathway. These surfaces can, in turn, be further functionalized with ligands such as antibodies or aptamers targeting PSMA, and/or cell-penetrating peptides to allow for receptor-mediated endocytosis of the cancer cells that can selectively take up the particles [107]. Such endosomal escape abilities are also required for sequestered siRNA in order to ensure complex formation with the RISC followed by sequence-dependent mRNA degradation within the cytosol after delivery of siRNA. Besides siRNA, other genetic materials such as microRNA (miRNA) 5 plasmid DNA, CRISPR components and antisense oligonucleotides are delivered via AuNPs. Gene therapy in conjunction with chemotherapy or photothermal therapy through multifactorial AuNP platforms should produce greater anticancer potency.

### 8.3. Combination Therapy Approaches

With two or more therapeutic schemes within a single nanoping, combination therapy using AuNPs offers an excellent opportunity to improve therapeutic efficacy in prostate cancer. Aside from antitumor studies including “traditional” chemotherapeutic agents or radio-therapy (radiotherapy) and hormone therapy monotherapies, these approaches are inherently complex with respect to mechanisms of drug resistance(s), systemic toxicity profiles, and incomplete tumor regression. While the targeted delivery and controlled release capabilities of AuNP have largely addressed these challenges, they can also be targeted to enable combination therapies via synergistic actions of guest molecules. Chemotherapeutic methods combined with PTT comprise one of the most commonly used methods. In addition to packing chemotherapeutic drugs such as docetaxel, AuNPs generate heat after NIR irradiation. Hyperthermia improves drug diffusion into the more advanced areas of solid tumors, breaks down solid tumor vasculature, increases pore size in cell membrane permeability and also sensitizes neoplastic population to apoptosis secondary to chemotherapy exposure, leading to greater cytotoxicity than either treatment modality independently [108].

The second strategy is AuNP-mediated radiosensitization during radiotherapy. AuNPs are characterized by high atomic numbers, which not only increases their radiation dose deposition but also enhances the production of ROS in the surrounding environment. Ultimately, combination with cluster-chemotherapy drugs or gene-silencing agents amplifies the DNA damage and inhibits the repair pathways responsible for genomic maintenance, leading to improved destruction of tumor cells. Combination therapy with either chemotherapy or phototherapy has also been investigated in several studies. Additionally, AuNPs could administrate siRNA, which results in co-delivery of resistance-associated genes with anticancer drugs to restore sensitivity and improve therapeutic efficacy against multidrug-resistant cancer. In like manner, gene silencing can be combined with photothermal or photodynamic therapy to produce mechanistic tumor ablation through heat and oxidative stress and pathway suppression [109].

The therapeutic approach could also be integrated into AuNP-based systems that target the androgen receptor pathway in second-generation castration-resistant prostate cancer. Moreover, theranostic multifunctional AuNP systems would serve the co-delivery of imaging (e.g., CT or photoacoustic imaging) moieties and therapeutic agents, providing a single construct for real-time observation of treatment response. Taken together, combination therapy involving gold nanoparticles offers heterotypic synergistic anticancer effect with decreased drug dose and decreased systemic toxicity, providing a future direction for precision and multimodal prostate cancer nanomedicine.

### 8.4. Mechanism of Controlled and Sustained Release

The controlled and sustained release mechanism is one of the honest design features of AuNP-mediated drug delivery systems in prostate cancer therapy, aiming to achieve the maximum required therapeutic concentration at the site of action with minimum systemic toxic weight burden and a minimal number of administrations [110]. In contrast to classical formulations that deliver drugs rapidly and indiscriminately, AuNP platforms can be tailored for both localized and time-dependent drug delivery. A more widely used approach is based on the surface functionalization of biodegradable polymers (PEG, chitosan or PLGA). These polymeric coatings act as diffusion barriers, releasing the drugs over time. The thickness of polymeric layers, their molecular weight and the degree of crosslinking can all be tuned to control release kinetics in a manner aimed at providing sustained therapeutic exposure. Another approach is based on conjugation through covalent linkers containing cleavable groups. pH-complementary, responsive redox or enzyme-releasable bonds to the AuNPs surfaces are used for drugs’ conjugation. PH-labile linkers, which are hydrolyzed under mildly acidic conditions present in tumor microenvironments or within endosomal/lysosomal compartments, facilitate specific drug release intracellularly. Likewise, redox-responsive drug release by disulfide bond cleavage cn be employed at elevated GSH (intracellular) levels. A site-dependent activation is tailored by the tumor-associated proteases to cleave the enzyme sensitive linkers. Release systems that are activated by either heat or light offer even more meticulous control. In the case of photothermal-based systems, AuNPs absorb NIR light to generate localized heat that either displaces polymer coatings or disrupts drug–nanoparticle interactions for on-demand therapeutic release. This might enable outside control of treatment timing and dosage [111].

**Figure 6:**
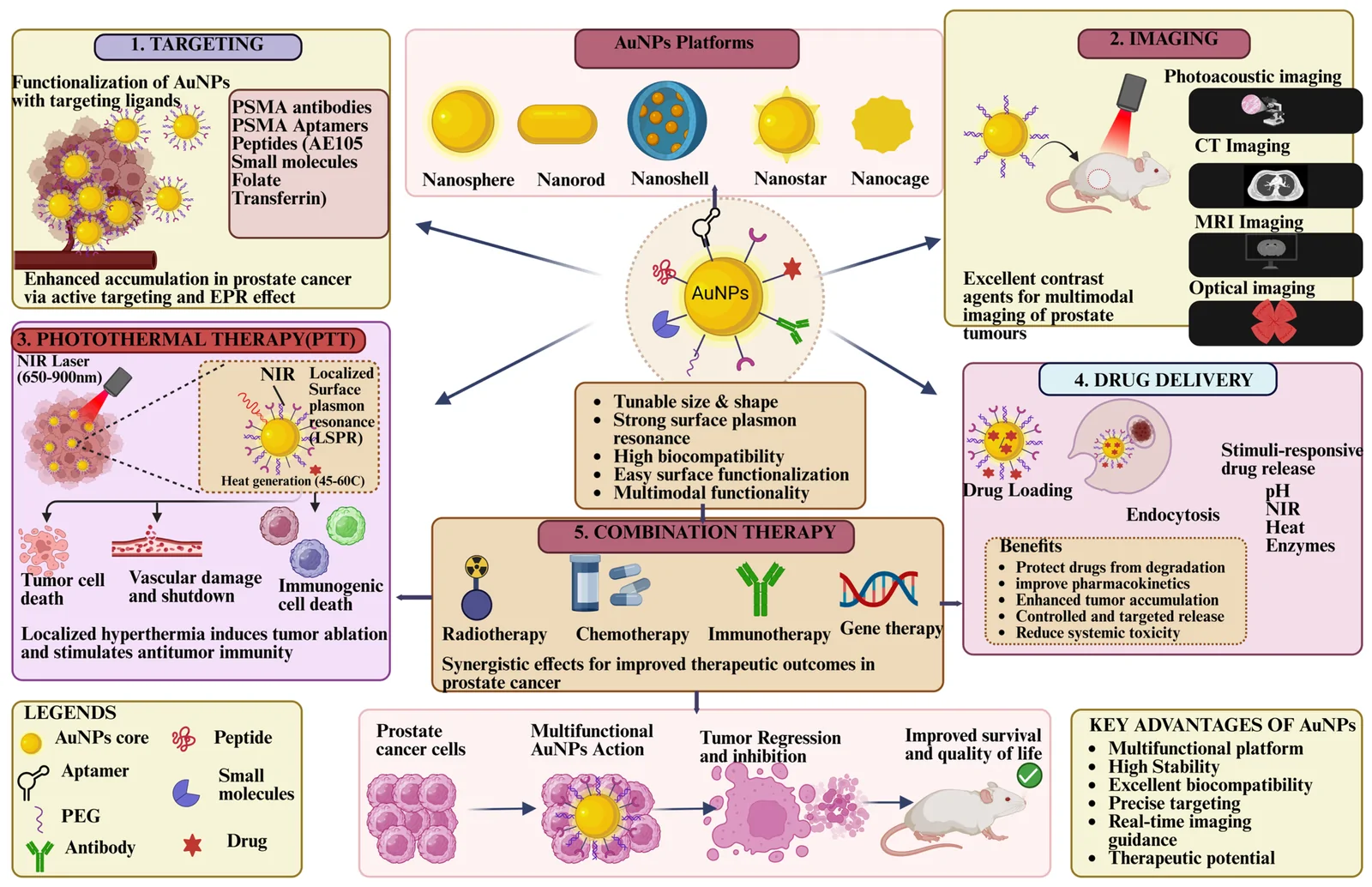
Schematic representation of multifunctional roles of AuNPs in prostate cancer. Created in BioRender. Hani, U. (2026) https://BioRender.com/zyqzqrw (accessed on 14 June 2026).

Moreover, layer-by-layer assembly techniques enable the provision of more than one type of therapeutic agent to be loaded in a sequential fashion so as to enable sustained and/or biphasic release profiles. The latter is of special relevance in combination therapy approaches where one drug sensitizes tumor cells for subsequent cytotoxicity induced by a second drug (Figure 6). Thus, controlled and sustained release approaches lead to favorable pharmacokinetics (thus reduced peak associated toxicity), enhanced tumor heterogenetic accumulation of drugs, and prolonged perfusion-phase concentrations within cells (Table 2). The incorporation of release and bioavailability into these advanced regimes represents an additional refinement of existing gold nanoparticle-based systems for treatments in prostate cancer with resultant enhanced therapeutic performance and safety profile.

## 9. Preclinical and Clinical Studies

The application of AuNPs in PC therapeutics has only been reported so far in preclinical and clinical research. In vitro and preclinical studies of AuNP-based systems have also been assessed in prostate cancer cell lines (e.g., LNCaP, PC-3 and DU145) as well as preclinically tested in vivo under xenograft or orthotopic conditions. These studies have shown that the use of AuNPs as drug carriers, radiosensitizers or photothermal agents can lead to higher cellular uptake, induction of apoptosis, inhibition of tumor growth and improved efficacy. Docetaxel-loaded AuNPs and PSMA-targeted nanoconjugates and stimuli-responsive platforms can significantly inhibit tumor growth while reducing systemic circulation toxicity. Further, AuNP-directed photothermal therapy and the enhancement of radiotherapy resulted in a significant reduction of tumors in animal studies, confirming their multimodal therapeutic efficacy.

AuNP-based biosensors and imaging agents have exhibited a very good sensitivity in detecting prostate cancer-specific biomarkers (PSA, PSMA etc.) in both laboratory and translational studies, which confirms the application of AuNP-based solutions in diagnostic scopes. Imaging-prepared AuNP formulations showed increased contrast resolution in CT and photoacoustic imaging in a preclinical setting. Many of the gold nanoparticle platforms found in clinical studies are at an early stage of clinical evaluation. So far, several gold-based nanotherapeutics and nanoshell-mediated photothermal systems have been developed in clinical translation and have shown acceptable safety and tolerability profiles, along with initial efficacy in Phase I trials of solid tumors. Further studies with larger sample sizes are ongoing to optimize biocompatibility, biodistribution control, long-term safety evaluations and regulatory standardization; large-scale clinical trials for prostate cancer are still in process but not yet widespread. Although promising results have surfaced, finding as to large-scale reproducible synthesis, long-term toxicity screening and pharmacokinetics in humans will be needed for clinical translation, which needs to be followed by regulatory approval pathways. In summary these studies provide a strong preclinical rationale for the use of therapeutic and diagnostic properties of gold nanoparticles in prostate cancer; nonetheless, safety profiles and efficacy as well as standardization of clinical protocols should be developed before this promising nanotechnology advances to clinical scenarios [127].

### 9.1. In Vitro Studies

Using prostatic AuNP, and understanding the mechanistic effects and their therapeutic utility in this setting in vitro is paramount in ensuring safe use as well as reliable assessment of therapeutic efficacy. The most common prostate cancer studies are performed under cytotoxic and/or therapeutic stress in well-characterized human prostate cancer cell lines of different disease types, namely LNCaP (androgen-dependent) and PC-3/DU145 (hydrogen-independent). Cytotoxicity, cellular uptake, apoptosis induction, oxidative stress generation and changes of gene expression induced by AuNPs treatment are assessed in controlled laboratory studies [128].

The anticancer cytotoxicity of free drugs and AuNP-conjugated formulations have been evaluated by several in vitro assays, such as MTT, XTT, Alamar Blue or CCK-8 to analyze cells’ viability after drug treatment. AuNPs-based nanocarriers are generally more effective anticancer agents owing to their manner of modulation of cellular internalization and sustained drug release. Flow cytometry and fluorescence microscopy determine nanoparticle uptake and intracellular localization, demonstrating receptor-mediated endocytosis using targeting ligands such as PSMA antibodies or peptides [129]. Various mechanistic studies in vitro evaluating apoptosis pathways have been performed using Annexin V/PI staining, induction of caspases activity assays and mitochondrial membrane potential assays; Western blot analysis was conducted for the Bax, Bcl-2 and p53 proteins. Many tests conducted in combination with radiotherapy or photothermal therapy indicate that AuNPs significantly promote ROS, causing G0/G1 and G2/M phase cell cycle arrest after exposure to AuNPs.

For applications such as gene delivery, in vitro transfection efficiency and gene silencing are confirmed by qPCR quantification (quantitative PCR assay) and protein expression analysis to validate the activity of the siRNA or miRNA. Hemocompatibility and biocompatibility assays are also used to assess safety profiles. Nonetheless, in vitro work provides valuable early information on efficacy, mechanism of action, effective targeting and toxicity that can guide subsequent in vivo and clinical studies involving gold nanoparticles as therapeutics in PC.

### 9.2. In Vivo Animal Models

Animal studies are scientifically obligatory for the characterization of pharmacokinetics, biodistribution and therapeutic efficacy and safety of AuNPs-based systems against prostate cancer. The most frequently implemented designs are xenograft models in which human prostate cancer cell lines (LNCaP, PC-3 or DU145)/genes are propagated subcutaneously into immunocompromised mice (nude or SCID mice); this permits the quantification of tumor growth inhibition, as well as nanoparticles accumulation via the EPR effect and the determination of targeted delivery approaches such as PSMA-functionalized AuNPs. Tumor volume, survival analysis and histopathological studies are commonly used to assess a therapeutic response [130].

These orthotopic models, in which prostate cancer cells are instilled directly into the mouse prostate gland, generate a more clinically relevant tumor microenvironment. The ultimate goal is humanized mouse models capable of faithfully recapitulating tumor progression, angiogenesis and metastasis that are better than the subcutaneous xenografts that have been utilized for the drugs in use today. AuNPs’ biodistribution and therapeutic performance in orthotopic systems are far more representative of treatment effectiveness. Besides using orthotopic models, metastatic models are also examined to study the therapeutic effects of AuNPs-based therapy in advanced prostate cancer. These impact models enable evaluation of tumor metastasis to either bone or lymph nodes, two commonly observed metastatic sites in prostate cancer.

The in vivo distribution and targeting of nanoparticles within the tumor can be monitored by imaging-based techniques including CT, photoacoustic imaging, fluorescence imaging and MRI, in addition to pharmacokinetic studies of half-life for circulation, and expulsion pathways. In hematological analysis, the serum biochemistry and histology of major organs provide evidence of toxicity assessment, confirming by this that the intended test material showed no adverse reaction in the host. Collectively, these preclinical animal models are valuable for confirming therapeutic responses and dosing schedules, assessing long-term safety parameters and converting gold nanoparticle therapies from bench-top studies into bedside applications in the context of prostate cancer [131].

### 9.3. Status of Clinical Evaluation and Translation

Although the clinical use of AuNP-based systems in prostate cancer therapy is still limited, we see plenty of encouraging results and this may further foster its development. Extensive preclinical data has also demonstrated significant therapeutic potential across drug delivery, radiosensitization, photothermal therapy and diagnostic imaging that has poised it for clinical evaluation. Some of the most distinguishing clinical developments in gold nanotechnology have been performed with silica/gold nanoshell platforms for surgeries. The system consists of near-infrared (NIR) light–responsive gold nanoshells and their application for selective photothermal ablation of solid tumors. AuNP-mediated photothermal exposure analysis was found to be feasible, and the tumor destruction was found to be localized without recrudescence upon analysis in early phase studies among prostate cancer patients that showed their acceptable safety profile, directly exhibiting a translational potential of this approach.

In addition to photothermal platforms, gold nanoparticles have shown promise as radiosensitizers for clinical application with the aim of increasing radiation dose delivery in tumors and reducing the damage on surrounding tissues. Researchers have summarized the current state of recent efforts in the development of radiosensitizing AuNP systems, a class that remains almost universally at preclinical or translatory prototype level; their conclusions are still compelled by a sound physical basis for dose enhancement and thus warrant continued exploration. Diagnostic applications are closer to translational incorporation [132].

Although steps are being taken in the right direction, there are still major barriers to clinical adoption. These include reproducible large-scale manufacturing, long term biodistribution and toxicity review, immune compatibility, regulatory harmonization and cost effectiveness. Genotoxicity, tumorigenicity and the potential long-term accumulation of nanoparticle-based therapeutics in organs (e.g., liver/spleen) through pharmacokinetics, and clearance mechanisms need to be characterized before regulatory approval can be obtained (Table 3). While gold nanoparticle-based therapeutics for prostate cancer are still largely in early-phase clinical development, the overall translational momentum is strong. Their continuous improvement of safety profiles (specificity and multifunctional capacity) is expected to foster their early use in precision oncology and multimodality cancer therapy approaches.

## 10. Toxicity, Biocompatibility, and Safety Concerns

Clinical application of AuNPs in treating prostate cancer must be explored rigorously with respect to their toxicity, biodistribution and long-term effects as they are relatively biocompatible, chemically inert vehicles [76]. Improving stability, reducing opsonization, prolonging circulation time and decreasing immune recognition can be achieved by polymer surface modification with biocompatible polymers like PEG. In some cases, however, the repeated administration of PEGylated nanoparticles can induce anti-PEG immune responses [138].

### 10.1. Long-Term Toxicity

A large number of both in vitro and in vivo studies have shown that, when appropriately engineered, AuNPs are quite low in acute toxicity relative to many other metallic nanoparticles. However, while the effects of short-term exposure may be negligible, over time there is still potential for it to yield biological consequences. In some experimental models, AuNPs produced persistent oxidative stress, mitochondrial dysfunction and lysosomal impairment, inflammatory responses, and changes in cellular metabolism. Such effects may be more related to the particle size, surface charge and coating materials rather than the gold core.

The reduced size causes a larger surface area with increased chemical reactivity which may elicit enhanced binding to intracellular proteins, nucleic acids and organelles. In contrast, larger nanoparticles are associated with decreased cellular toxicity but prolonged tissue retention.

### 10.2. Organ Accumulation and Biodistribution

After their systemic administration, AuNPs distribute throughout the organism based on their intrinsic chemical properties. Nanoparticle sequestration is principally mediated by the mononuclear phagocyte system (MPS) or reticuloendothelial system (RES). As a result, the liver and spleen are preferentially where AuNPs get distributed due to large outlets of resident macrophages (Kupffer cells for the liver; splenic macrophages).

While hepatic accumulation is seldom acutely toxic, the chronic deposition that occurs after repeat dosing may affect liver function and exacerbate oxidative stress in specific scenarios. Likewise, the prolonged exposure of splenic-stored bacteria to immune cells may likewise influence immune homeostasis. AuNPs have also been reported in smaller quantities in the lungs, kidneys, lymph nodes and bone marrow, as well as the brain under particular experimental conditions.

### 10.3. Biodegradation Limitations

In contrast, metallic gold is extremely resistant to chemical degradation in physiological environments unlike biodegradable polymeric nanoparticles. This remarkable stability leads to long circulation as well as sustained action, but also prevents complete removal of the drug from the biological system after therapy. Consequently, AuNPs may stay in lysosomes and reticuloendothelial tissues for long time after a cell uptake.

While recent work indicates that nanoscale Au particles could partially degrade in acidic lysosomal conditions, bulk gold nanoparticles are known to be largely biostable. Non-degradable nanoparticles remain in the organism, where they could cause cumulative toxicity after several administrations, especially in patients that need continuous treatment. To overcome these drawbacks, recent studies have used biodegradable hybrid nanoplatforms, ultrasmall renal-clearable gold nanoclusters and surface coatings responsive to stimuli that can achieve gradual disassembly of nanoparticles post therapy [139].

### 10.4. Clearance Pathways

The elimination of AuNPs from the body is strongly size- and shape-dependent. Size-dependent renal secretion nanoparticles that have a size smaller than about 5–8 nm are likely to be filtered by the glomerular membrane and then excreted in urine. In comparison, larger nanoparticles are mostly eliminated by hepatobiliary pathways, in which they are internalized into Kupffer cells and removed gradually via bile to gastrointestinal tract.

The clearance kinetics can be corrected through surface modification by PEGylation, zwitterionic coatings and hydrophilic polymer coatings that reduce the formation of a protein corona and enable prolonged retention in the systemic circulation. However, prolonged circulation can also enhance healthy tissue exposure to nanoparticles, thus necessitating compromise between therapeutic efficacy and quick elimination post-treatment.

Recent breakthroughs have aimed at synthesizing ultrasmall or biodegradable AuNPs with comparable efficacy in therapy while allowing quick excretion via the kidneys after drug administration and less long-term retention within tissues.

## 11. Challenges

Despite the potential of AuNPs in many facets of prostate cancer diagnosis and therapy, significant scientific and technological hurdles remain before AuNPs can be utilized clinically. An important issue is the reproducibility and standardization of the synthesis of nanoparticles. The influence of various factors in nanomedicine activity, such as changes in size, shape, surface chemistry and ligand density, on a nanoparticle can lead to drastic changes in biological activity as well as therapeutic efficacy and toxicity [140]. The repeated dose regime safety requires routine pharmacokinetic profiling, clearance studies and prolonged toxicity assessments.

Another challenge is tumor heterogeneity and targeting efficiency. In some patient populations, variable expression of the biomarker, i.e., PSMA, could limit actively targeted systems. Moreover, biological barriers such as tumor microenvironment, endosomal trapping, and immune clearance can limit the delivery efficiency. Current research explores ways to enhance tumor penetration, facilitate escape from endosomes and avoid detection by the immune system. The complexity of approval is also due to the multiple functionalities of AuNPs platforms, particularly the theranostic applications (simultaneously imaging and therapeutic) [141].

## 12. Future Perspectives

This approach offers a closer perspective towards personalized nanomedicine and precision. These systems, along with molecular profiling, may enable patient-specific targeted approaches. However, these advances are expected to result in the construction of multi-responsive and multifunctional nanoplatforms that can synergistically trigger chemotherapy, gene therapy, and photothermal therapy while simultaneously demonstrating real-time imaging for better therapeutic effects. Thus, the development of AI-assisted coffers (such as biomimetic coatings like cell membrane cloaking) for engineering novel biocompatible and biodegradable gold nanoclusters that complement safety and targeting efficacy can be carried out in parallel to these advances [142,143]. Furthermore, the adoption of scalable production technologies and standardization of safety evaluation with clinical trials are critical to translate research into practice. Thus, even if there are great aspects such as research multimodalities and novel technologies, gold nanoparticle-based methods may constitute promising candidates for precision prostate cancer diagnosis and therapy in the future.

## 13. Conclusions

AuNPs and their various physicochemical, biological and functional properties are all discussed for the potential use in therapeutics against PC. As such, their unique optical properties arising from surface plasmon resonance along with controllable size and morphology, high surface functionalization potential and relatively low toxicity contribute to their multifaceted role as therapeutic and diagnostic nanoplatforms. AuNPs are also being used for targeted delivery of drugs, gene silencing, radiosensitization and photothermal and photodynamic therapy in addition to enhanced imaging and biosensing of prostate cancer. Moreover, their integration as theranostic systems allows for simultaneous diagnosis and therapy as well as the dynamic monitoring of therapeutic effects in vivo, paving the way towards precision-based oncotherapy. However, large-scale synthesis and reproducibility, long-term safety, biodistribution limitations and the lack of regulatory standardization pose significant hurdles for successful clinical translation. Their potent application will be pivotal for the further optimization of NP design, along with extensive toxicological evaluation of engineered surfaces and well-controlled clinical trials. This review article describes these nanoparticles as a promising versatile platform for next-generation prostate cancer therapy, and their enhanced understanding in biological systems provides the basis for design strategies that would facilitate drug delivery or co-administration of therapies as part of personalized-medicine approaches.

## Figures and Tables

**Table 2:** Gold nanoparticle-mediated drug delivery systems in prostate cancer.

AuNP Platform/Modification	Drug/Therapeutic Agent	Targeting Strategy	Key Research Findings	Model System	Reference
PEGylated AuNPs	Docetaxel	Folate receptor targeting	~96% drug encapsulation efficiency; IC_50_ reduced from ~8.5 μg/mL (free docetaxel) to ~3.2 μg/mL; PC-3 viability decreased to ~40%.	PC-3 cell line	[112]
D2B antibody-conjugated AuNPs	Therapeutic cargo/imaging agents	PSMA antibody targeting	~3-fold higher binding affinity and receptor-mediated uptake in PSMA-positive PC-3-PSMA cells compared with non-targeted AuNPs.	PC-3-PSMA cells	[113]
PSMA-targeted PEG-AuNPs	Pc4 photosensitizer	PSMA ligand targeting	~4-fold higher tumor accumulation; ~70–80% tumor volume reduction after photodynamic therapy.	PC3pip tumor model	[114]
Phosphatidylserine-capped AuNPs	Phosphatidylserine	Biomimetic targeting	~65% apoptotic cells and significant DNA fragmentation compared with untreated control.	In vitro prostate cancer cells	[115]
PEGylated AuNP nanocarriers	Paclitaxel	Passive targeting (EPR effect)	IC_50_ decreased from ~10.4 μM (free paclitaxel) to ~4.6 μM in PC-3 cells; enhanced cytotoxicity in DU145 cells.	PC-3/DU145 cells	[116]
AuNP-liposome hybrid nanoparticles	Doxorubicin	Passive tumor targeting	~2.3-fold higher intracellular drug accumulation; IC_50_ reduced from ~6.8 μM to ~2.9 μM.	In vitro prostate cancer model	[117]
PSMA-ligand functionalized AuNPs	Docetaxel	Active targeting via PSMA	~2.8-fold higher uptake in PSMA-positive cells; tumor growth inhibition of ~65% in vivo.	PC-3 tumor model	[118]
Au nanorods	Photothermal agents	NIR-mediated tumor targeting	Tumor temperature reached ~55 °C; ~75% tumor growth inhibition after NIR irradiation.	Murine prostate tumor model	[119]
PEG-AuNPs	siRNA	Gene silencing targeting	~80% target gene knockdown; ~55% reduction in PC-3 cell proliferation.	PC-3 cells	[120]
AuNP-chitosan nanocarriers	Curcumin	Passive uptake	IC_50_ reduced from ~15.2 μM (free curcumin) to ~6.4 μM; apoptosis increased to ~60%.	In vitro prostate cancer cells	[121]
Aptamer-functionalized AuNPs	Doxorubicin	Aptamer-mediated targeting	IC_50_ decreased from ~5.7 μM (free doxorubicin) to ~2.1 μM; reduced toxicity in non-target cells.	LNCaP cells	[122]
Gold nanoclusters	Anticancer drugs	Passive targeting	~36% renal clearance within 24 h; reduced liver accumulation and systemic toxicity.	Animal tumor models	[123]
PEG-coated AuNPs	Radiotherapy sensitizers	Tumor accumulation	Dose enhancement factor (DEF) ~1.5–2.0; ~50% greater tumor regression compared with radiotherapy alone.	Prostate tumor xenografts	[124]
AuNP-polymer conjugates	Mitoxantrone	Passive delivery	IC_50_ reduced from ~7.9 μM (free drug) to ~3.5 μM; sustained release over 48–72 h.	DU145 prostate cancer cells	[125]
Multifunctional AuNP theranostic platform	Drug + imaging agent	Targeted theranostics	~2-fold higher tumor accumulation and ~60% tumor growth inhibition with simultaneous imaging capability.	Preclinical prostate tumor models	[126]

**Table 3:** Clinical trials of gold nanoparticles in prostate cancer.

Gold Nanoparticle Platform	Clinical Application	Clinical Trial (NCT)	Phase	Primary Endpoint	Secondary Endpoints	Major Findings	Limitations	Implications for Future Research	Reference
AuroShell^®^ (gold–silica nanoshells)	Focal photothermal ablation of localized prostate cancer	AuroLase Therapy for Focal Ablation of Prostate Tissue (NCT02680535)	Completed	Evaluate safety and successful focal ablation of prostate tissue	MRI-guided treatment accuracy, adverse events, quality of life, urinary and sexual function	Demonstrated selective laser-mediated tumor ablation with preservation of surrounding healthy tissue and acceptable safety profile.	Single-arm study, relatively small sample size, short follow-up, absence of randomized control group.	Larger randomized multicenter trials with long-term oncological and functional outcome assessment are required.	[133]
AuroShell^®^ (gold nanoshells)	Photothermal therapy for solid tumors	Study of AuroLase Therapy for Tumor Ablation (NCT00848042)	Completed (Phase I)	Determine safety and feasibility of nanoparticle-mediated photothermal therapy	Maximum tolerated laser dose, treatment response, adverse events	Demonstrated feasibility of nanoparticle-assisted thermal ablation with favorable safety profile.	Early-phase study involving heterogeneous tumor types and limited statistical power.	Future studies should evaluate efficacy in larger disease-specific patient cohorts.	[134]
AuroShell^®^ (gold nanoshells)	Focal treatment of prostate cancer	Nanoparticle-Directed Laser Therapy for Prostate Cancer (NCT01679470)	Terminated	Evaluate safety and technical feasibility	Tumor response, procedural success, adverse events	Trial was terminated before completion; therefore, efficacy could not be established.	Early termination, inadequate patient recruitment, no efficacy analysis possible.	Improved trial design, optimized recruitment strategies, and multicenter collaboration are necessary.	[135]
AuroShell^®^ (gold nanoshells)	MRI-guided nanoparticle-assisted focal therapy	Nanoparticle-Directed Photothermal Therapy for Prostate Tumors (NCT04240639)	Recruiting/Ongoing	Safety and effectiveness of focal photothermal ablation	Progression-free survival, MRI response, quality of life, urinary function	Results are pending; study aims to validate clinical efficacy and long-term safety.	Ongoing trial; no published efficacy outcomes currently available.	May provide higher-level clinical evidence supporting image-guided AuNP-mediated focal therapy.	[136]
CYT-6091 (PEGylated AuNP-TNF-α; Aurimune^®^)	Nanoparticle-mediated cytokine delivery in advanced solid tumors	Phase I Trial of CYT-6091 (NCT00356980)	Phase I Completed	Evaluate safety, dose-limiting toxicity, and maximum tolerated dose	Pharmacokinetics, tumor response, systemic toxicity	Demonstrated safe systemic administration of TNF-α at doses exceeding those achievable with free TNF-α, with reduced systemic toxicity.	Phase I design, heterogeneous tumor population, no control arm, limited efficacy assessment.	Supports further evaluation of AuNP-based cytokine delivery in combination with chemotherapy, immunotherapy, or radiotherapy.	[137]

## Data Availability

No new data was created or analyzed in this study. Data sharing is not applicable to this article.
